# Camouflaging attenuated *Salmonella* by cryo-shocked macrophages for tumor-targeted therapy

**DOI:** 10.1038/s41392-023-01703-1

**Published:** 2024-01-10

**Authors:** Leyang Wu, Zengzheng Du, Lin Li, Liyuan Qiao, Shuhui Zhang, Xingpeng Yin, Xiaoyao Chang, Chenyang Li, Zichun Hua

**Affiliations:** 1 The State Key Laboratory of Pharmaceutical Biotechnology and Department of Neurology of Nanjing Drum Tower Hospital, School of Life Sciences and The Affiliated Hospital of Nanjing University Medical School, Nanjing University, Nanjing, 21008 Jiangsu China; 2 Nanjing Generecom Biotechnology Co., Ltd, Nanjing, 210023 China; 3 Changzhou High-Tech Research Institute of Nanjing University and Jiangsu TargetPharma Laboratories Inc, Changzhou, 213164 Jiangsu China; 4 School of Biopharmacy, China Pharmaceutical University, Nanjing, 210023 Jiangsu China

**Keywords:** Drug development, Cell delivery

## Abstract

Live bacteria-mediated antitumor therapies mark a pivotal point in cancer immunotherapy. However, the difficulty in reconciling the safety and efficacy of bacterial therapies has limited their application. Improving bacterial tumor-targeted delivery while maintaining biosafety is a critical hurdle for the clinical translation of live microbial therapy for cancer. Here, we developed “dead” yet “functional” *Salmonella*-loaded macrophages using liquid nitrogen cold shock of an attenuated *Salmonella typhimurium* VNP20009-contained macrophage cell line. The obtained “dead” macrophages achieve an average loading of approximately 257 live bacteria per 100 cells. The engineered cells maintain an intact cellular structure but lose their original pathogenicity, while intracellular bacteria retain their original biological activity and are delay freed, followed by proliferation. This “Trojan horse”-like bacterial camouflage strategy avoids bacterial immunogenicity-induced neutrophil recruitment and activation in peripheral blood, reduces the clearance of bacteria by neutrophils and enhances bacterial tumor enrichment efficiently after systemic administration. Furthermore, this strategy also strongly activated the tumor microenvironment, including increasing antitumor effector cells (including M1-like macrophages and CD8+ Teffs) and decreasing protumor effector cells (including M2-like macrophages and CD4+ Tregs), and ultimately improved antitumor efficacy in a subcutaneous H22 tumor-bearing mouse model. The cryo-shocked macrophage-mediated bacterial delivery strategy holds promise for expanding the therapeutic applications of living bacteria for cancer.

## Introduction

Living bacterial therapeutics have reshaped the landscape of tumor immunotherapy,^1,2^ with roots tracing back to the late 19th century when Dr. William Coley harnessed heat-inactivated *Streptococcus pyogenes* and *Serratia marcescens* to create Coley’s toxin for cancer treatment.^3^ In patients, a tumor size reduction was successfully observed with bacteria-based therapeutics. Although the mechanism underlying their function in mediating antitumor therapeutics is not clear, it is speculated that the immunogenicity of bacteria can direct the immune response to tumors. A range of bacteria, such as *Salmonella typhimurium*, *Listeria monocytogenes*, *Escherichia coli*, and *Bifidobacterium bifidum*, have shown preclinical antitumor efficacy.^1,2^ These microorganisms are also referred to as oncolytic bacteria. Their facultative anaerobic characteristics make them prefer the low-oxygen environment inside tumors, allowing them to establish and proliferate effectively within tumors (1000 times, even higher, more concentrated of strains in the tumor microenvironment than in healthy tissue) and subsequently activate intratumoral immune cells through a series of different strategies.^4–8^ However, the injection of heterologous microorganisms invariably triggers a rapid immune response in the body, leading to discomfort and potential adverse effects.^8–10^ Toxicity to the host from direct administration of live bacteria has been shown to limit tolerable doses and efficacy.^1,2,11^ Ideal bacterial therapies for cancer should minimize toxic effects caused by off-target or antigenic stimulation of bacteria to ensure high biocompatibility.

A series of efforts have been undertaken to enhance the biosafety of bacterial therapies, such as genetic knockouts, to reduce strain immunogenicity.^1,12^ This approach yielded the well-known classical oncolytic bacteria attenuated *Salmonella typhimurium* VNP20009. Purine deficiency and decreased lipopolysaccharide modification come from the deletion or mutation of the *purl* and *msbB* genes in the genome of the VNP20009 strain. This strategy, at the expense of some of the efficacy, partially enhanced the biocompatibility of these strains, but there were still observable toxic side effects, such as systemic inflammatory response, and led to a reduction in bacterial colonization of tumors.^11^ Another strategy is to camouflage bacteria with coatings on their surfaces,^13^ e.g., alginate,^14^ polydopamine,^15^ and cell membranes.^16,17^ However, the shedding of modifiers resulting from the natural and persistent motility and proliferative activity of bacteria remains a potential safety concern. Enhancing biocompatibility without compromising bacterial intratumoral colonization and tumor immune activation ability remains the most important challenge for microbe-mediated antitumor therapy.

In recent years, functional polymer materials for the loading, transfer and release of anticancer drugs have been widely studied, and cell-mediated drug delivery systems have become a promising research field. These systems employ various cell types, including erythrocytes and immune cells (e.g., macrophages, neutrophils, T lymphocytes, and natural killer cells), as drug delivery carriers due to their dynamic roles in biological systems.^18–20^ Because these cells are “self” materials, cell-mediated drug delivery systems offer several advantages over conventional methods, including prolonged circulation times, enhanced tissue targeting precision, enhanced ability to cross physiological barriers, and improved biocompatibility.^21–23^ Among them, macrophages are found in all tissues and play a vital role in the innate immune system’s defence against a variety of infections and cancers.^24^ Macrophages have been shown to govern a wide range of disorders, including cancer, infection, and autoimmune disease. At the same time, macrophages can phagocytize and load nanoparticles, and after activation at the lesion site, the carrier macrophages discharge cellular contents, including drugs.^21,22,25^ As a result of their high loading capacity and tumor targeting enrichment, macrophages have sparked widespread interest as natural delivery vehicles. We previously reported a pioneering strategy involving macrophage-mediated tumor-targeted delivery of *Salmonella typhimurium* VNP20009 (abbreviated VNP).^25^ To live following macrophage-mediated phagocytosis, *Salmonella* can create a number of self-protective strategies and use macrophages as a natural “Trojan horse” in the body.^26,27^ Nevertheless, primary macrophages that work as “Trojan horse” are difficult to culture and have limited proliferative capacity,^28^ which leads to limited clinical translational potential for strategies based on the delivery of bacteria by such cells. This contradiction is highlighted more acutely when high doses of cells are needed to meet therapeutic needs. Ideal carrier macrophages should be simple to prepare, rapidly accessible, safe and controllable.

**Fig. 1:**
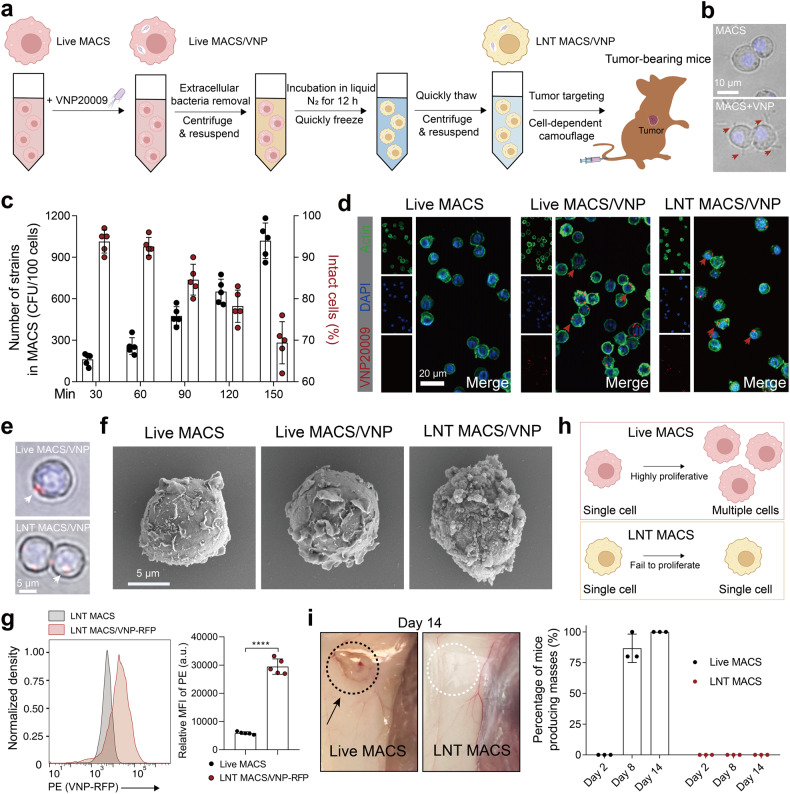
Preparation and characterization of cryo-shocked macrophages loaded with VNP strains. **a** Schematic diagram of the LNT MACS/VNP cell preparation process. **b** Bright-field pictures of macrophages (MACS) cocultured with VNP strains (MACS + VNP). Hoechst marks nuclei (blue). Red arrows indicate the VNP strains. Scale bar = 10 μm. **c** Changes in the number of live VNP strains loaded intracellularly in MACS and the percentage of morphologically intact MACS over the time the cells were cocultured with the strain (*n* = 5). **d** Fluorescence images of Live MACS, Live MACS/VNP cells and LNT MACS/VNP cells. FITC-phalloidin marks actin (green); DAPI marks nuclei (blue). Red arrows indicate the intracellular VNP-RFP strains. Scale bar = 20 μm. **e** Bright-field pictures of Live MACS/VNP and LNT MACS/VNP cells. Hoechst marks nuclei (blue). White arrows indicate the VNP strains (strain expressing red fluorescent protein). Scale bar = 5 μm. **f** Scanning electron microscopy (SEM) was used to observe the morphology of different cells. Scale bar = 5 μm. **g** Relative mean fluorescence intensity (MFI) of LNT MACS and LNT MACS/VNP-RFP is represented. a.u., arbitrary unit. **h** Schematic of the proliferative activity comparison of Live MACS and LNT MACS. **i** Comparison of the in vivo proliferative activity of Live MACS and LNT MACS (right). Percentage values are summarized from three independent experiments (*n* = 5 mice per group per independent experiment). Representative anatomically observed images of mass generation are shown (left). The area inside the dotted ellipse represents the site of inoculation. The data are reported as the mean ± SD. All data are representative of two independent experiments. **** *p* < 0.0001

In this work, we developed a liquid nitrogen cryo-shocked engineered macrophage cell line preloaded with VNP strains that can be acquired quickly and readily and stored stably for safer and more convenient bacteria-mediated tumor immunotherapy. Specifically, the macrophage cell lines RAW264.7 was first coincubated with VNP strains, and VNP-loaded macrophages (Live MACS/VNP) were obtained. Then, the cells were cryo-shocked with liquid nitrogen to obtain liquid nitrogen-treated MACS/VNP cells (LNT MACS/VNP) (Fig. 1a). LNT MACS/VNP cells retain their original structure and tumor enrichment ability for tumor-targeted delivery of VNP strains, followed by activating the tumor immune microenvironment. At the same time, due to the packaging of macrophages, a delivery mode similar to “Trojan horses” is formed, which reduces the exposure of heterogeneous bacteria and minimizes toxic side effects caused by strains. In addition, cold shock treatment of macrophage cell lines achieves compatibility between rapid cell acquisition and biocompatibility, which will effectively extend the application of this strategy.

## Results

### Preparation and characterization of cryo-shocked macrophages loaded with VNP strains

To generate VNP-loaded macrophages, the VNP strain was cocultured with the classical mouse macrophage cell line RAW264.7, which is routinely employed for drug delivery.

The strain actively infects the cells, while macrophages are also able to phagocytose the strain and finally successfully “load” VNP strains (Fig. 1b). Gentamicin was used to remove extracellularly adherent bacteria without affecting intracellular bacterial activity^25,29^ (Supplementary Fig. 1a). The number of live strains loaded in macrophages increased with coincubation time (Fig. 1c). The survival of strains in macrophages results from the self-protection mechanisms of the strains, including boosting bacterial antimicrobial peptide resistance related-genes expression and preventing the development of intracellular lysosomal proteins.^26,27^ However, the integrity of macrophages was disrupted with strain loading over time (Fig. 1c, Supplementary Fig. 1b), which may result from prolonged strain stimulation.^30^ Overall, a coculture time of 60 min was selected because of both high loading of live strains within cells (257 ± 27 strains per 100 cells) and high cell integrity (>90%) (Fig. 1c). Moreover, intracellular *Salmonella* induces macrophages toward an M1-type proinflammatory phenotype (Supplementary Fig. 2), which means greater tumor cell killing potential. Fluorescence imaging was used to offer an intuitive visualization of the intracellular strain (Fig. 1d). Ultimately, we obtained VNP-loaded macrophages (Live MACS/VNP).

Subsequently, the collected Live MACS/VNP cells were snap-frozen using liquid nitrogen to obtain liquid nitrogen-treated MACS/VNP cells (LNT MACS/VNP) (Fig. 1a). The VNP-RFP strain (an engineered VNP strain expressing red fluorescent protein (RFP)), was used in subsequent experiments for better visualization. Fluorescence microscopy (Fig. 1d, e) and scanning electron microscopy (SEM) (Fig. 1f) observations showed that neither strain loading nor cryo-shock significantly affected the overall integrity of the cells. It is not difficult to speculate that this strategy achieves effective protective properties, as bacterial xenobiotics (e.g., flagella, bacterial surface polysaccharides) are no longer directly exposed to the organism’s environment. Engineered VNP-RFP strains inside the LNT MACS/VNP cells were also detected with the aid of flow cytometry and reconfirmed the intracellular loading of cells to bacteria (Fig. 1g). Furthermore, LNT MACS may be used to load two separate modified strains with various target genes (including VNP-RFP and VNP-GFP), meaning that this cell-based camouflage delivery method can deliver at least two therapeutic genes (Supplementary Fig. 3a). There was a slight reduction in macrophage volume after cryo-shock (Supplementary Fig. 3b). The immortalized live RAW264.7 macrophage cell line demonstrated rapid proliferation in vitro, enabling the swift preparation of strain-loaded macrophages using these cells (Fig. 1h, Supplementary Fig. 3c). It is important to emphasize that the LNT MACS described here lost pathogenicity, and subcutaneous injection of these cells into mice no longer resulted in heterogeneous mass generation on day 14 after injection, in contrast to the rapid mass generation observed with live cells within 5 days (Fig. 1i, Supplementary Fig. 3c, d). Finally, we obtained LNT MACS/VNP cells loaded with VNP strains, maintaining their overall structure and eliminating pathogenicity.

### Intracellular VNP strains can be released by LNT MACS/VNP cells

**Fig. 2:**
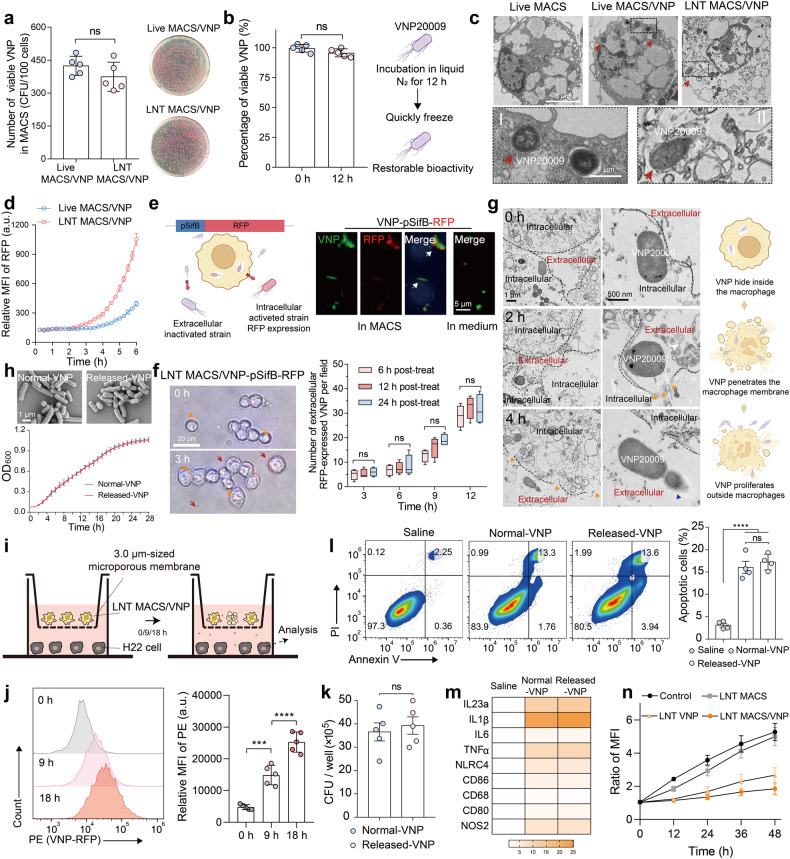
Intracellular VNP strains can be released by LNT MACS/VNP cells and maintain their original biological activity. **a** Changes in the number of intracellular live strains of Live MACS/VNP and LNT MACS/VNP cells (left) (*n* = 5). Representative coated plates are shown (right). **b** Survival ratios of VNP strains after liquid nitrogen cold treatment for 12 h (*n* = 5). Schematic diagram of changes in strain activity before and after cryo-shock treatment (right). **c** Transmission electron microscopy (TEM) was used to observe the morphology of different cells. Red arrows indicate the intracellular strains. Scale bar = 5 μm (normal field) and 1 μm (magnified field). **d** The change in RFP fluorescence intensity of Live MACS/VNP-RFP and LNT MACS/VNP-RFP cell culture plates was monitored in real-time (*n* = 3). **e** Schematic diagram of the use of VNP-pSifB-RFP (red fluorescent protein, RFP) to examine bacterial release by MACS (left). The VNP-pSifB-RFP strain was only activated and expressed RFP intracellularly because of the pSifB promoter. Fluorescent-field pictures of VNP-psifB-RFP strains in MACS or LB medium (right). Activated VNP-psifB-RFP strains (red); Salmonella-specific antibodies mark VNP (green); DAPI marks nuclei (blue). Scale bar = 5 μm. **f** Bright-field pictures of the release of intracellular VNP-pSifB-RFP strains from LNT MACS/VNP-pSifB-RFP cells (left). Hoechst marks nuclei (blue). Orange arrows indicate the intracellular strains, and red arrows indicate the extracellular strains. The cells were cultured in medium supplemented with trace amounts of gentamicin. Scale bar = 20 μm. Five visual fields were chosen at random from three liquid nitrogen treatment time groups (6/12/24 h post-treatment) at different time points to count extracellular RFP-expressing bacteria (right). **g** The release of intracellular VNP strains from LNT MACS/VNP cells was observed with the aid of TEM (left). The white curve shows the movement of the strain from intracellular to extracellular. The orange arrow indicates the notch on the surface of LNT MACS cells, which may result from intracellular strain motility. Blue arrows indicate the extracellularly proliferating bacteria. Scale bar = 1 μm (normal field) and 500 nm (magnified field). Schematic diagram of intracellular strain release (right). **h** The bacterial morphology (top) and growth curves (bottom) of Normal-VNP and LNT MACS-released VNP in LB liquid medium at 37 °C (*n* = 3). **i** Schematic of indirect coculture of LNT MACS/VNP cells with H22 tumor cells by using a 3.0-μm Transwell chamber. Bacteria can traverse chambers of this pore size. **j** Comparisons of the relative mean fluorescence intensity (MFI) of H22 tumor cells in (**i**) after coculturing for different times. **k** H22 cells were infected with different VNP at an MOI of 100 for 1 h and the number of internalized VNP was determined by plating the cell lysates on LB solid plates (*n* = 5). **l** Representative FACS analysis of Annexin V and propidium iodide (PI) staining after H22 cells were incubated with different strains at an MOI of 100 for 4 h, and the quantification analysis of the percentage of apoptotic cells (Annexin V^+^ cells) is shown on the right (*n* = 4). **m** Antitumor M1-type macrophage-related gene expression levels were detected by real-time PCR after coculturing different strains with M0-type macrophages for 6 hours. **n** Proliferation of H22 tumor cells after incubation with LNT VNP, LNT MACS, or LNT MACS/VNP, or not. The cell nuclei dye Hoechst was used to detect changes in cell number. The data are reported as the mean ± SD. All data are representative of two independent experiments. *** *p* < 0.001; **** *p* < 0.0001; n.s. not significant

We then assessed the impact of rapid cryo-shock treatment with liquid nitrogen on intracellular bacterial viability. Interestingly, the intracellular strain maintained the same level of activity as before cryo-shocking, indicating that the loading rate of VNP in macrophages was not altered by LNT treatment. (Fig. 2a). Additionally, the untreated free VNP strain demonstrated robust survival following cryo-shock treatment. Even under continuous exposure to liquid nitrogen for up to 48 h, the bacterial survival rate remained at ~95% once suitable conditions were restored (Fig. 2b, Supplementary Fig. 4a). This is understandable because bacteria can achieve cold-temperature resistance by regulating membrane composition and cold shock protein expression.^31,32^ Transmission electron microscopy (TEM) revealed intact strains within the cells, including live macrophages and cryo-shocked macrophages (Fig. 2c). The strain-loaded macrophages also maintained high cell integrity after cryo-shocking, consistent with previous results (Figs. 1d–f, 2c). The effective survival of intracellular strains (Fig. 1c), the preservation of high cellular and intracellular bacterial integrity following cryo-shock treatment (Fig. 2c), and the notable resistance of the strains to low-temperature damage (Fig. 2a, b, Supplementary Fig. 4a) collectively support the feasibility of cryo-shocked cells as a viable strategy for bacterial camouflage carriers.

We next explored whether these intracellular strains can be effectively released from LNT MACS/VNP cells, similar to live macrophages.^30^ Significant strain proliferation was detected within 3 h of incubation in LNT MACS/VNP-RFP cells, which preceded Live MACS/VNP-RFP cells (Fig. 2d, Supplementary Fig. 4b, c). The release of strains may result from sustained proliferation and motility of intracellular strains. LNT MACS/VNP exhibits an earlier strain release compared to Live MACS/VNP, which might be attributed to an impaired ability to inhibit and kill intracellular strains in the former. Next, we constructed the engineered strain VNP-pSifB-RFP. The promoter sifB (pSifB) is a *Salmonella*-specific regulatory expression promoter that is initiated by the intracellular microenvironment, such as low pH and low phosphate.^33,34^ Fluorescence images showed that the VNP-pSifB-RFP strain selectively expressed RFP after entering macrophages but not in regular media. VNP-pSifB-RFP specifically expressed RFP after entering macrophages, while the strain cultured in normal medium did not (Fig. 2e). Notably, strains expressing RFP were also observed outside the LNT MACS/VNP cells over time (Fig. 2f), and these fluorescence-expressing strains were undoubtedly released from within the cells. To avoid interference caused by self-proliferation of activated VNP-pSifB-RFP strains, trace amounts of gentamicin were added to the medium to reduce bacterial proliferation but not kill them (Supplementary Fig. 1a). Observations by TEM visualized the release of intracellular strains more directly (Fig. 2g). These bacteria were liberated from LNT MACS and exhibited rapid proliferation outside the cell over time (Fig. 2d, g). Moreover, the acidic conditions that simulated the tumor microenvironment^18^ had no significant influence on strain release or proliferation (Supplementary Fig. 4d). These results again confirmed that the intracellular strains could be released from cells and proliferate extracellularly. The released-VNP strains did not exhibit significant differences in growth rate, strain morphology or ability to infect tumor cells compared to normal VNP strains (Fig. 2h–k). In addition, the released-VNP strains demonstrated their effectiveness in inducing apoptosis in tumor cells and promoting the polarization of M0 macrophages to the antitumor M1 phenotype (Fig. 2l, m). Thus, we can reasonably conclude that these intratumorally released-VNP strains have the potential to facilitate tumor regression by directly killing tumor cells and reshaping the tumor microenvironment.^1,2^ Additionally, compared to LNT MACS cells, LNT MACS/VNP cells showed higher levels of reactive oxygen species (ROS), which could kill tumor cells, in their lysate (Supplementary Fig. 5). The high ROS level and the ability of bacteria to induce tumor cell death partly explain why LNT MACS/VNP cells are significantly more effective at inhibiting the proliferation of tumor cells in vitro than LNT MACS and LNT VNP cells (Fig. 2n). In conclusion, these findings suggest that intracellular strains of cryopreserved cells maintain their biological activity, enabling delayed release and consistent antitumor efficacy even after release.

### LNT MACS protects the VNP strain from clearance and promotes its accumulation in tumors

**Fig. 3:**
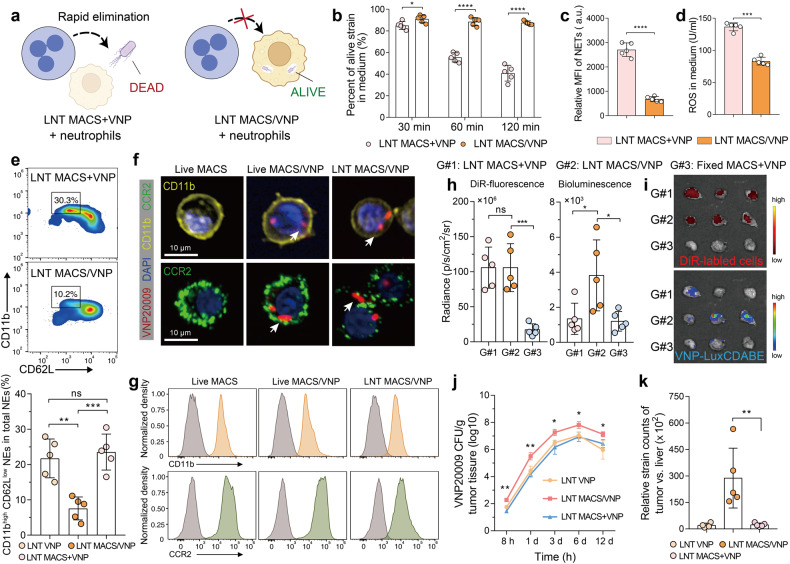
LNT MACS protects the VNP strain from clearance and promotes its accumulation in tumors. **a** Schematic diagram of LNT MACS/VNP cells camouflaging intracellular strains to evade neutrophil-mediated bacterial clearance. **b** Changes in the percentage of live strains in the medium after primary peritoneal neutrophils were cocultured with LNT MACS + VNP and LNT MACS/VNP for different times. **c, d** Comparison of NETs and ROS levels produced by neutrophils in the medium after different treatments in (**b**) at 60 min. **e** A representative flow cytometric plot (top) and a bar comparison chart (bottom) of the change in the percentage of activated neutrophils (CD11b^high^ CD62L^low^) in peripheral blood total neutrophils 1 h after different treatments (*n* = 5). **f** CD11b and CCR2 expression in Live MACS, Live MACS/VNP and LNT MACS/VNP cells analyzed by confocal microscopy. White arrows indicate the intracellular strains. Scale bar = 10 μm. **g** Representative flow cytometry of CD11b and CCR2 in different cells. **h** Fluorescence and bioluminescence intensities of the tumors of the indicated groups are shown (*n* = 5). DiR-labeled cells and VNP-LuxCDABE strains were used. **i** Fluorescence images of tumors isolated 8 h after different treatments in (**h**). **j** The relationship between time postinjection and bacterial number within the tumor (*n* = 4 or 5). **k** The tumor:liver ratios of bacterial CFU per gram were calculated on the basis of recovered CFU from extracted organs on day 6 (*n* = 5). The data are reported as the mean ± SD. All data are representative of two independent experiments. * *p* < 0.05; ** *p* < 0.01; *** *p* < 0.001; **** *p* < 0.0001; n.s. not significant

Neutrophils in the body are the most important line of defense against microorganisms because of their potent antimicrobial activity. However, *Salmonella* are able to avoid neutrophil elimination by using macrophages as a natural haven in vivo, as these bacteria can survive for a long time in macrophages.^25,35^ We speculated that bacteria within LNT MACS could similarly evade neutrophil clearance. To verify this speculation, we cocultured activated primary peritoneal neutrophils with LNT MACS + VNP and LNT MACS/VNP cells and detected the change in the number of total viable bacteria at different time points (Fig. 3a). The results showed that the percent of live VNP strains within the simple mixed group of neutrophils, LNT MACS and LNT VNP was significantly lower compared to the coincubation between neutrophils and LNT MACS/VNP cells (Fig. 3b). Close to 90% of the bacteria remained active in the LNT MACS/VNP group after 60 min of cocultivation with neutrophils, while less than 60% remained active in the LNT MACS + VNP group (Fig. 3b). Correspondingly, higher levels of neutrophil extracellular traps (NETs) and ROS, which neutrophils use to capture and kill free bacteria, respectively,^36^ were detected in the latter culture medium (Fig. 3c, d). The percentage of activated neutrophils^35,37^ in the peripheral blood, which are stimulated by heterologous strains of bacteria and possess bactericidal properties, was higher in the LNT MACS + VNP group (~23.5%), while there was significant remission in the LNT MACS/VNP group (~7.6%) (Fig. 3e). These results demonstrate that LNT MACS-mediated camouflage avoids neutrophil activation and neutrophil-caused strain killing, thereby contributing to the sustained enrichment of the strain in the tumor. Less neutrophil activation in peripheral blood also implies higher biosafety.

Macrophages are able to achieve targeted enrichment at tumor sites through a range of strategies, which correlate, at least in part, with the expression of CD11b and CCR2 proteins. They are two key receptors that work for integrin-mediated cell adhesion and chemokine receptor-mediated cell chemotaxis.^38,39^ Most of the proteins expressed by MACS cells were detectable in LNT MACS cells (Supplementary Fig. 6a). We next examined the expression of CD11b and CCR2 proteins on the surface of the different cells. Western blotting showed that high levels of CD11b and CCR2 proteins were still present in LNT MACS/VNP cells (Supplementary Fig. 6b). Fluorescence microscopy and flow cytometry assays more visually demonstrated that CD11b and CCR2 proteins were yet significantly present on the surface of LNT MACS/VNP cells (Fig. 3f, g, Supplementary Fig. 6c).

We then evaluated the intratumor enrichment ability of LNT MACS/VNP cells. After intravenous administration of DiR-labeled different cells to tumor-bearing mice, a similar significant enrichment of labeled LNT MACS/VNP cells and LNT MACS cells inside the tumor was detected after treatment, while it was reduced in paraformaldehyde-fixed cells (Fig. 3h, i, Supplementary Fig. 7a, b). This demonstrates the positive role of surface proteins for LNT MACS cells in achieving intratumoral enrichment. The similar time-course profile of the strain release number and its tumor site accumulation number imply a possible logical relationship (Supplementary Fig. 7c). Notably, an increased lactate concentration accelerated the proliferation of intracellular strains, followed by the faster release of intracellular bacteria in vitro, consistent with previous reports^40^ (Supplementary Fig. 7d, e). This suggests that upon LNT MACS/VNP cell arrival at the tumor, the high lactate level may assist in specific intracellular strain release. The surface integrin and chemokine receptors, including CD11b and CCR2 (Fig. 3f, g, Supplementary Fig. 6), coupled with the stronger entrapment effect of disorganized and tortuous micrometer-diameter tumor capillaries, may facilitate LNT MACS/VNP cell accumulation in the tumor lesion.^39^ Thus, it is understandable that significantly higher intratumoral strain titers and lower strain titers in normal organs were detected in the LNT MACS/VNP cell group than in the LNT VNP group (Fig. 3j, Supplementary Fig. 8a–f). There was no significant difference in the distribution of strains corresponding to the LNT VNP group and the LNT MACS + VNP group (Fig. 3j, Supplementary Fig. 8a–f). The results, which include the higher titer of intratumor strains in the LNT MACS/VNP cell group compared to the LNT VNP group and the exclusion of potential strain growth differences (Fig. 2h), reconfirmed the effectiveness of LNT MACS-mediated strain delivery. The released strains colonizing the tumor proliferated rapidly due to the unique tumor microenvironment (Fig. 2h, Fig. 3j). The tumor:liver ratios of bacterial CFUs per gram on day 6 in the LNT MACS/VNP group were increased by ~12.5 times compared with those in the LNT MACS + VNP group (Fig. 3k). Compared to the limited tumor targeting of direct administration of strains or simple mixing of strains and cells, LNT MACS-based strain postloading delivery promoted their accumulation in the tumor site (Fig. 3h–k, Supplementary Fig. 7a). These results suggest that the higher biosafety as well as the higher strain titer within the tumor achieved by LNT MACS/VNP cells may be a result of LNT MACS-mediated protection and intratumoral enrichment.

### LNT MACS/VNP reduces strain-induced biotoxicity

**Fig. 4:**
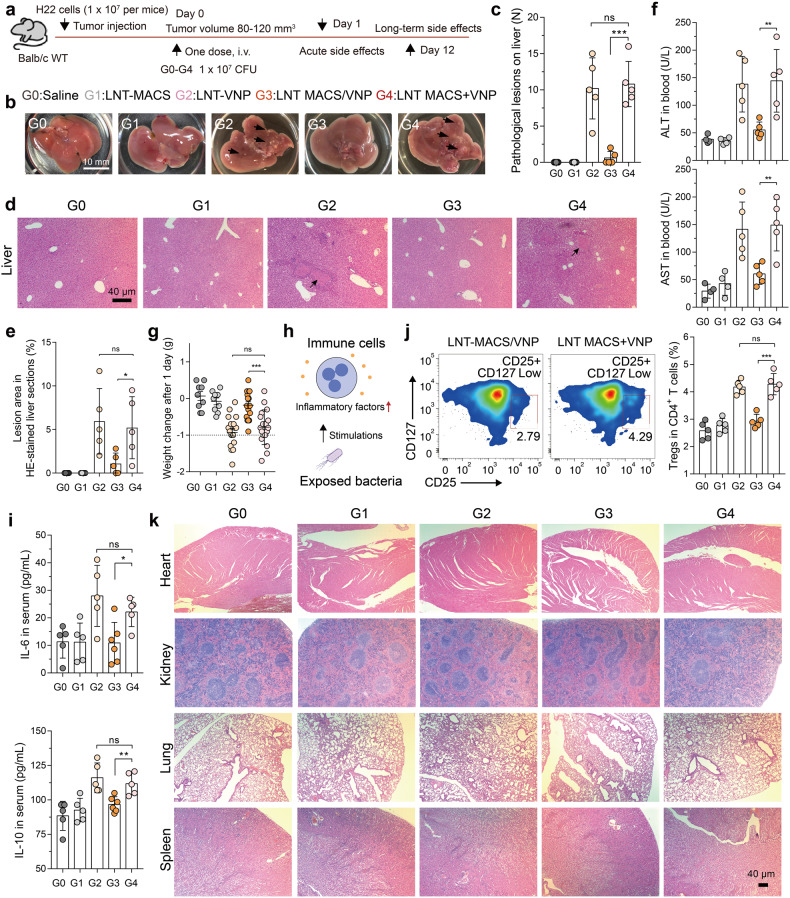
LNT MACS/VNP reduces strain-induced biotoxicity. **a** Schematic diagram of the safety assessment, tumor targeting and therapy assay of different groups (G0-G4) on H22 tumor-bearing mice. **b** Representative pictures of hepatic inflammatory lesions in each group of mice 1 day after administration. Black arrows indicate the site of pathological liver lesions. Scale bar = 10 mm. **c** Bar graph comparing the number of hepatic inflammatory lesion points in each group in (b) (*n* = 5). **d** Close-up view of representative H&E staining showing liver injury (black arrow) 1 day after treatment. Scale bar = 40 µm. **e** Five visual fields in (d) were randomly selected to count the area of the lesion. **f** Serum alanine transaminase (ALT) and aspartate transaminase (AST) levels 1 day after different treatments (*n* = 4 or 5). **g** Changes in body weight 1 day after different treatments (*n* = 9 or 18). **h** Schematic diagram of bacterial stimulation of immune cells to produce inflammatory factors. **i** Detection of IL-6 (top) and IL-10 (bottom) concentrations in the peripheral blood of H22 tumor-bearing mice 1 day after different treatments (*n* = 5 or 6). **j** Change in the percentage of Tregs (CD25^+^ CD127^Low^ cells) among CD4^+^ T cells from the peripheral blood 1 day after different treatments. A representative flow cytometric plot is shown (*n* = 5). **k** Close-up view of representative H&E staining of heart, kidney, lung and spleen sections (scale bar = 40 µm). The data are reported as the mean ± SD. All data are representative of two independent experiments. * *p* < 0.05; ** *p* < 0.01; *** *p* < 0.001; **** *p* < 0.0001; n.s. not significant

Normally, bacterial immunogenic surface antigens normally cause fast, even excessive, immune activation after entering the body, which is followed by harmful side effects to the organs.^9^ We speculate that the camouflage protection of LNT MACS may attenuate these effects induced by the bacteria. To test this hypothesis, we administered LNT MACS/VNP cells *via* tail vein injection in H22 tumor-bearing mice, a commonly used model for bacterial tumor targeting and biosafety studies,^1^ and tested for acute side effects 1 day after administration (Fig. 4a). Administration of a single liquid nitrogen-treated VNP strain (LNT VNP) induced significant hepatic inflammation with 4–15 lesions per liver in mice, while LNT MACS/VNP treatment showed effective alleviation with less than two lesions per liver (Fig. 4b, c). Simple mixing of LNT MACS cells with LNT VNP strains (LNT MACS + VNP) did not alleviate the symptoms, suggesting that the improved biosafety resulted from intracellular loading and camouflage of strains by LNT MACS (Fig. 4b, c). The H&E-stained sections of liver regions from different groups also showed that VNP strains or a simple mixture of LNT MACS and VNP strains triggered lesions in tissue regions, which were effectively mitigated in the LNT MACS/VNP group (Fig. 4d, e). The levels of ALT/AST in serum and the extent of weight loss in the LNT MACS/VNP group were significantly lower than those in the LNT VNP group and the LNT MACS + VNP group (Fig. 4f, g), again indicating reduced liver injury in the former.

Exposure to bacterial flagella or polysaccharides stimulates immune cells to produce large amounts of inflammatory factors (Fig. 4h), which is often one of the key triggers of liver lesions,^9,10^ and there exists a correlation between the quantity of Treg cells present in the bloodstream and the degree of inflammation observed in vivo.^41^ Therefore, we examined the levels of representative inflammatory factors (including IL-6 and IL-10) and the CD4 + CD25+ CD127low Treg cell populations in peripheral blood after different administrations for 24 h. The upregulation of inflammatory factors as well as Treg cells in the blood triggered by the strain was no longer significant in LNT MACS/VNP (Fig. 4i, j). Furthermore, no significant damage to normal organs was found in the LNT MACS/VNP group (Fig. 4k). There were also no detectable chronic toxicities after treatment, even in the liver (Supplementary Fig. 9a–d). This may be due to gradual clearance of the strain and the liver’s natural self-repair mechanism over time. These results suggest that LNT MACS-based camouflage protection indeed moderates the toxic side effects induced by bacterial therapies compared to single strain administration.

### LNT MACS/VNP enhances antitumor efficacy and activates antitumor immunity

**Fig. 5:**
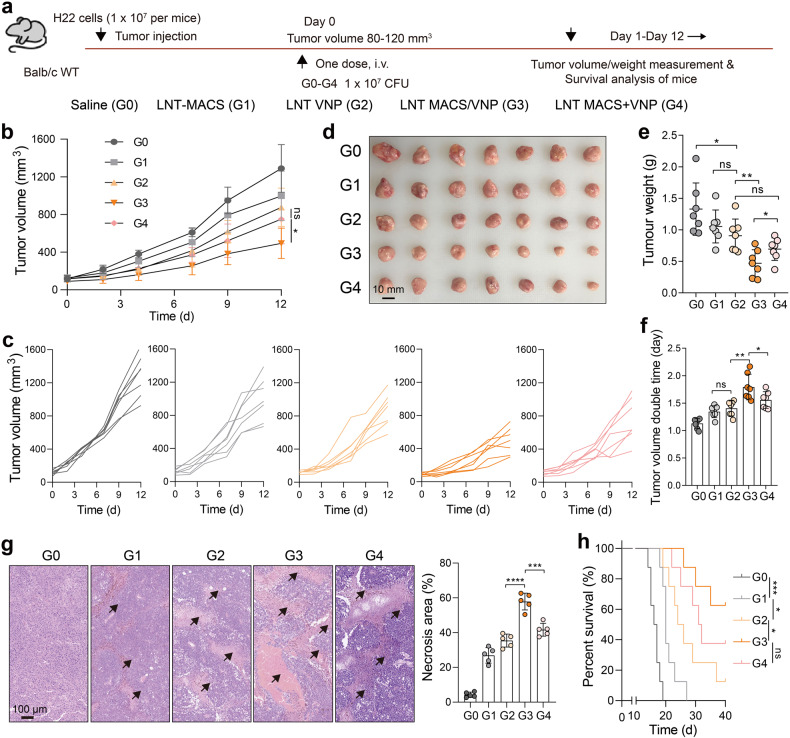
LNT MACS/VNP cells effectively inhibit H22 tumor growth. **a** Schematic diagram of the safety assessment, tumor targeting and therapy assay of different groups (G0-G4) on H22 tumor-bearing mice. **b** Tumor growth profiles after different treatments (*n* = 7). **c** Tumor growth curves for each mouse in (b). Tumors were photographed (**d**) and weighed (**e**) 12 days after administration in (**b**). Scale bar = 10 mm. **f** Comparison of tumor volume doubling time in different groups in (**b**). **g** Representative H&E staining images of the tumor (left), and five visual fields were randomly selected to count the area of the necrotic regions (right). Black arrows indicate the necrotic regions. Scale bar = 100 µm. **h** Survival curve of mice treated as described in (**a**). The mice were killed when they reached a humane endpoint. Data are reported as the mean ± SD. *p ≤ 0.05; **p ≤ 0.01; *** *p* < 0.001; **** *p* < 0.0001; n.s. not significant

To test the anticancer efficacy of this LNT MACS-mediated bacterial camouflage redelivery, we administered LNT MACS/VNP cells to H22 subcutaneous tumor-bearing mice in a single intravenous injection. The same dose of injected LNT MACS, LNT VNP and a simple mixture of the two (LNT MACS + VNP) was used as a control (Fig. 5a). Tumor growth curves indicated a slight antitumor efficacy of single LNT MACS, which was similarly detected on live macrophages^25^ and may be derived from the tumor cell killing effect of ROS (Figs. 5b, 2n, Supplementary Fig. 5). In comparison to both the LNT VNP group and the LNT MACS + VNP group, the LNT MACS/VNP group demonstrated a more pronounced tumor suppression effect (Fig. 5b, c). The tumors of different groups were photographed and weighed to more visually demonstrate the differences in anticancer efficacy. The tumor weight of the mice receiving LNT MACS/VNP treatment was only 35.4%, 44.6%, 51.2% and 67.7% of that of the mice treated with saline, LNT MACS, LNT VNP and LNT MACS + VNP on day 12 after administration, respectively (Fig. 5d, e). The LNT MACS/VNP group achieved a significantly longer tumor volume doubling time (1.79 days) than the saline (1.12 days), LNT MACS (1.34 days), LNT VNP (1.40 days) and LNT MACS + VNP (1.55 days) groups (Fig. 5f). In contrast, more necrotic areas were observed within the tumors of mice treated with LNT MACS/VNP (Fig. 5g). Moreover, the LNT MACS/VNP group also effectively prolonged the survival of mice compared to the single VNP group and the LNT MACS + VNP group. Survival rates exceeding 40 days were observed in 60% of mice treated with LNT MACS/VNP, compared to no more than 40% in the other groups. (Fig. 5h). It is understandable that LNT MACS/VNP achieved enhanced anticancer efficacy, as indicated by its higher intratumoral strain titers (Fig. 3j, k).

**Fig. 6:**
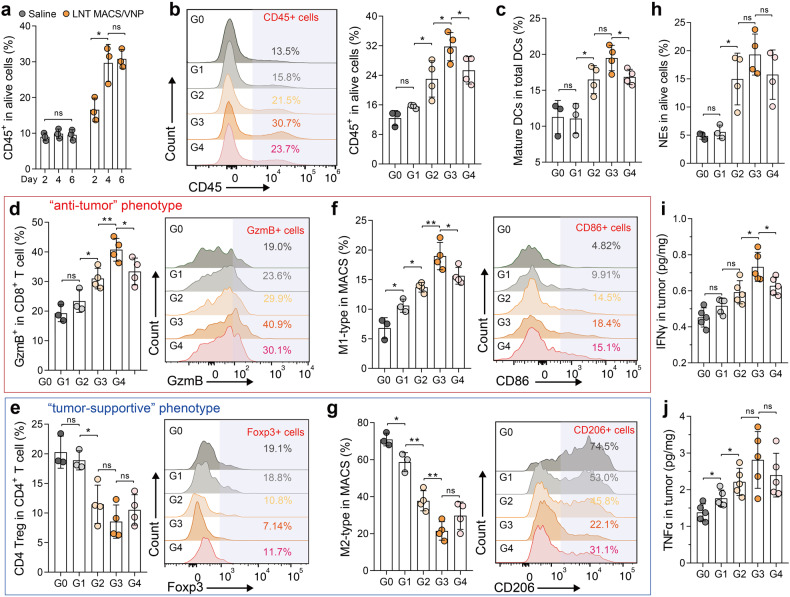
LNT MACS/VNP cells activate antitumor immunity. **a** Changes in the tumor-infiltrating immune cell (CD45+) population on days 2, 4 and 6 after different treatments (*n* = 3). **b** A representative flow cytometric plot (left) and a bar comparison chart (right) of the change in the percentage of immune cells (CD45+) in tumors after different treatments (*n* = 3 or 4). **c** Change in the percentage of mature DCs (CD11c+ MHCII+ CD86+) in tumors on day 4 after different treatments (*n* = 3 or 4). A representative flow cytometric plot (right) and a bar comparison chart (left) of the change in the percentage of tumor-infiltrating lymphocytes, including CD8+ Teff (**d**) and CD4+ Treg (**e**), as well as M1-like (**f**) and M2-like (**g**) tumor-infiltrating macrophages on day 4 after different treatments (*n* = 3 or 4). **h** Change in the percentage of NEs (CD11b+ Ly6G+ F4/80-) in tumors on day 4 after different treatments (*n* = 3 or 4). Detection of IFNγ (**i**) and TNFα (**j**) concentrations in tumors 4 days after administration (*n* = 5). G0 Saline, G1 LNT MACS, G2 LNT VNP, G3 LNT MACS/VNP, G: LNT MACS + VNP. Data are reported as the mean ± SD. *p ≤ 0.05; **p ≤ 0.01; n.s. not significant

Tumor progression is always coupled with changes in the tumor microenvironment. Thus, we used flow cytometry to assess changes in the intratumoral immune microenvironment 4 days after therapy, which led to dramatically increased intratumoral immune cell numbers (Fig. 6a, Supplementary Fig. 10). The results showed that LNT VNP, LNT MACS/VNP, and LNT MACS + VNP all effectively promoted intratumoral immune cell infiltration, with LNT MACS/VNP obtaining the highest induction (~31.7%) (Fig. 6b). LNT MACS/VNP also promoted intratumoral DC maturation (CD86+ DCs) (Fig. 6c). Mature DCs are able to potently activate T cells,^42^ so it is understandable that the LNT MACS/VNP group exhibited significantly higher levels of Teff (GzmB+ CD8 T) cells within the tumors, showing a 2.11-fold increase compared to the saline group (Fig. 6d). Moreover, lower levels of Treg (Foxp3+ CD4 T) cells were observed, reaching only 42.2% of those in the saline group (Fig. 6e). Additionally, the percentage of M1-type macrophages, which are associated with antitumor activity, significantly increased (~2.79 times) after LNT MACS/VNP treatment compared to the saline group, while the proportion of M2-type macrophages, which have immunosuppressive effects, decreased (~0.30 times) (Fig. 6f, g). The infiltration of neutrophils within the tumor was also significantly elevated (Fig. 6h), which may be recruited by stimulated intratumoral macrophages and potentially contribute to the antitumor response.^36^ Intratumoral TNFα and IFNγ, two critical effector molecules in antitumor immunity, were elevated 2.04-fold and 1.62-fold, respectively, after LNT MACS/VNP treatment compared with saline treatment (Fig. 6i, j). These elevated proinflammatory factors may be derived from activated or recruited T cells, macrophages, neutrophils, etc. (Fig. 6d–h).^2^ Taken together, these results suggest that LNT MACS/VNP achieves more efficient antitumor therapy by activating antitumor immunity.

## Discussion

Here, we report for the first time the use of liquid nitrogen cold-shocked macrophages for tumor-targeted delivery of *Salmonella* and verify the feasibility, efficacy, and safety of this strategy. Normal macrophages obtained from mice are theoretically better delivery vectors, given their potentially superior in vivo tumor targeting ability. In fact, a series of studies have already confirmed the effectiveness of using autologous normal immune cells as delivery carriers for bacterial tumor targeting.^25,43,44^ However, primary macrophages have limited proliferative potential, and their culture conditions are stringent, making them expensive and challenging to use for general medical applications. This is a typical challenge faced by all other traditional cell therapies.^45^ To achieve the desired therapeutic effects, traditional cell-based cancer therapies or drug delivery methods require the injection of large quantities of cells (10^6^–10^7^ cells in mice or 10^7^–10^8^ cells in humans).^46–48^ This means longer preparation times and higher costs. Immortalized macrophage cell lines, which can proliferate indefinitely and are easy to culture, offer a feasible solution to the problem of cell sourcing. However, their use may raise pathogenicity problems. Thus, we aim to address these challenges by using “dead” but “functional” liquid nitrogen-treated macrophage cell lines (LNT MACS) to eliminate their pathogenicity while maintaining accessibility. This is crucial given that it is still challenging to swiftly and easily obtain enough and safe cells for conventional cell therapies. Similar techniques have demonstrated encouraging outcomes on tumor cells and stem cells,^49,50^ and immune cells may have a larger enrichment effect in tumors since they are naturally more sensitive and adherent to tumor cells as well as intratumor blood channels.

The method of bacterial delivery through intravenous or intraperitoneal injection, which is commonly employed in preclinical and clinical trials,^51^ exposes the bacterium to the host’s healthy immunological environment immediately. On the one hand, strains elicit a rapid immunological response, including processes such as neutrophil recruitment and activation,^52^ resulting in their rapid clearance. On the other hand, the enrichment of bacteria within the tumor after bacterial administration may be influenced by the complex interplay of tumor vasculature^53^ and immune cell trafficking^43^ rather than being solely attributed to active bacterium targeting. As a result, some strains will still be randomly transferred to normal organs, where they may cause inflammatory lesions.^54^ Our experiments also corroborated these findings, which included (1) the observation that direct contact between neutrophils and bacteria can trigger rapid bacterial clearance by neutrophils, often involving mechanisms such as NETs and ROS production, and (2) the finding that direct intravenous injection of VNP strains induces acute liver injury, although subsequent repair occurs. Striking the delicate balance between ensuring biosafety and optimizing antitumor efficacy, which is often influenced by changes in administered doses, has a direct impact on the clinical application of bacterial therapies.^11^ Although intratumoral injection significantly reduces the accumulation of VNP strains in the liver and spleen, it is undoubtedly not the most applicable mode of administration for tumor treatment and cannot be used for deep-seated tumors or metastatic lesions, greatly restricting its clinical translational applications. The approach of loading LNT macrophages has contributed to resolving these challenges, resulting in safer and more efficient intratumoral bacterial enrichment. Ultimately, a high bacterial titer in the tumor is achieved through the rapid growth of intracellularly released strains within the tumor.

Our results demonstrate that a single round of low-temperature shock treatment does not significantly affect the biological activity of most proteins, which is consistent with previous research,^50^ and it does not impact the biological activity of engineered bacteria. We assessed the proliferation and side effects of LNT MACS in vitro and in vivo. After liquid nitrogen–based cryo-shocked treatment, most proteins remain detectable in macrophage cell lines, including surface chemotactic factor receptors and integrins that determine their chemotaxis, adhesion and residency in tumors.^38,39^ Additionally, all mice treated with LNT cells did not exhibit significant side effects, and there were no records of abnormal mass growth within 14 days of LNT MACS inoculation compared to the live macrophage cell line RAW264.7, which formed a mass at the injection site within 5 days. More importantly, cryo-shock treatment did not affect the cell integrity, proliferation, or invasion ability of VNP strains. Furthermore, VNP strains can rapidly recover their activity after this cryo-shock treatment, allowing them to sense environmental changes and be rapidly released from vector cells, contributing to the success of this antitumor strategy. These results support the safety and effectiveness of the proposed strategy in our mouse model. However, considering potential clinical implications, the safety of LNT MACS/VNP cells should be thoroughly evaluated in various models beyond the H22 tumor model used in this study, including orthotopic tumor models and tumor metastasis models.^1,2^ The preservation of cell protein bioactivity and bacterial functionality suggests the possibility of synthetic biology modifications for the two key components within LNT MACS/VNP (LNT MACS and VNP). For example, to improve the intratumor enrichment ability of LNT MACS cells, macrophages can be engineered with chimeric antigen receptors.^47^ Furthermore, improved intracellular VNP strains can be developed for bacterial-mediated intratumoral protein drug delivery, such as anti-PD1 nanobodies, anti-CD47 nanobodies, and more.^25,55^ These developments hold the potential to enhance the anticancer efficacy of this therapy and warrant further investigation.

**Fig. 7:**
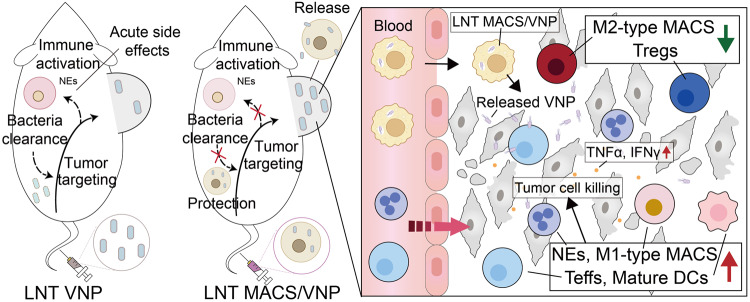
Schematic diagram of cryo-shocked macrophage-mediated tumor-targeted delivery of VNP strains. The protection of LNT MACS avoids neutrophil activation triggered by the exposed strain and enables intratumor-targeted release of intracellular strains with the help of macrophages. Strains released and proliferating within the tumor achieve effective tumor suppression by enhancing antitumor immune responses

Taken together, our findings represent the first evidence that cryo-shocked MACS/VNP cells can serve as “Trojan horses”, aiding in the accumulation of VNP strains within tumors. Liquid nitrogen shock eliminated the pathogenicity of the macrophage cell line while preserving the integrity of its cellular structure, allowing LNT macrophages to retain their effect enrichment toward tumor sites when used as drug carriers. Furthermore, liquid nitrogen shock had no impact on the activity and infectivity of the VNP strain, ensuring the preservation of its antitumor activity, which determines the effectiveness of this strategy. By limiting direct exposure of bacterial heterologous stimulation to the host environment during the treatment process, this technique enhances the biosafety and biocompatibility of bacteria-based cancer therapy. Targeted delivery by LNT macrophages further increases the bacterial titer within the tumor, helping to establish a more robust antitumor immune response and thus enhancing antitumor efficacy (Fig. 7). These findings open exciting possibilities for the development of safer and more potent bacterial-based anticancer treatments, contributing significantly to the evolving landscape of anticancer therapies.

## Materials and methods

### Cells and strains

The H22 mouse hepatoma cell line, RAW264.7 mouse macrophage cell line, and attenuated *Salmonella typhimurium* VNP20009 were preserved in our laboratory. Cells were cultured using Dulbecco’s modified Eagle medium (Gibco) supplemented with 10% fetal bovine serum (FBS, Gibco). All strains were cultured using LB solid or liquid medium. The VNP strain constitutively expressing RFP (VNP-RFP strain), green fluorescent protein (GFP) (VNP-GFP strain), bioluminescence (LuxCDABE) (VNP-Lux strain), and the VNP strain controllably expressing RFP with the aid of the sifB promoter (VNP-psifB-RFP strain) were all constructed as described previously.^33^ All plasmids used in this study were constructed using the ClonExpress Ultra One Step Cloning Kit V2 (Vazyme, C116, Nanjing, China). The constructed plasmids, including pJ23100-RFP, pJ23100-Lux, and psifB-RFP, were electrotransformed into VNP competent cells, with the transferred empty vector plasmids serving as blank control strains and denoted as VNP-NC. DH5α and BL21 competent cells were purchased from Vazyme. Mycoplasma contamination was excluded by a mycoplasma elimination reagent (Yeasen, 40607ES03, Shanghai, China).

### Preparation of VNP20009-competent cells

The protocol listed below was followed to prepare *Salmonella* competent cells. LB broth (1% peptone, 0.5% yeast extract, and 1% NaCl) was injected with a single colony chosen from the plate of activated attenuated *Salmonella* strains and then cultivated at 37 °C to an OD_600_ of 0.4–0.6. The bacterial liquid was centrifuged to capture the bacteria after being incubated in an ice bath for 15–30 min. The bacteria were collected by centrifugation at 4 °C and 5000 rev/min for 5 min and washed with precooled 10% glycerol three to four times. The bacteria were resuspended at a concentration of 3.5 × 10^8^ CFU (colony forming unit)/mL and used for electroporation.

### Preparation of liquid nitrogen-treated VNP-loaded macrophages (LNT MACS/VNP)

Macrophages were plated into 6-well plates at 5 × 10^5^ cells per well and cultured using antibiotic-free cell culture medium. Monoclonal strains were picked from the agar plates and activated overnight in LB liquid medium. Bacterial broth grown to log phase was centrifuged at 8000 rpm for 5 min, and the supernatant was removed and resuspended in sterile saline. After adjusting the OD_600_ to 1.0, the bacteria were added to 6-well plates plated with the cells described above (MOI 20) and incubated together for various times. To observe morphological changes in macrophages, cells were observed with the help of a microscope after staining cell nuclei with Hoechst (Solarbio, C0030, Beijing, China). Changes in the percentage of broken macrophages at different time points were recorded. Subsequently, the supernatant was removed and washed 2–3 times with sterile PBS and then incubated for 30 min using cell culture medium supplemented with 50 µg/ml gentamicin. Gentamicin was able to kill extracellular strains without a significant effect on intracellular strains.^25^ The culture supernatant was removed, and the sedimented cells were resuspended and washed 2–3 times with sterile PBS. Finally, the cells were collected to obtain live macrophages loaded with the VNP strain (Live MACS/VNP). To obtain liquid nitrogen cold-treated strain-loaded macrophages, the above obtained Live MACS/VNP cells were well mixed using serum-free cell lyophilization solution. Subsequently, the cell suspension was snap-frozen directly in liquid nitrogen and left to stand for 12 hours before being removed. Liquid nitrogen cold-treated strain-loaded macrophages (LNT MACS/VNP) were obtained. LNT MACS/VNP cells freshly obtained from liquid nitrogen are recommended to be incubated in culture medium at 37 °C for 20 min to initially restore intracellular strain activity before being used in subsequent studies.

### Morphology and activity of LNT MACS/VNP cells

To compare the morphological changes in macrophages before and after liquid nitrogen cold treatment, the above-prepared MACS, Live MACS/VNP, and LNT MACS/VNP cells were photographed using SEM and TEM.

For SEM, Live MACS/VNP and LNT MACS/VNP were prefixed in 2.5% isopropanol at room temperature for 2 h and washed with 0.1 M phosphate buffer (pH 7.4). Then, the samples were suspended and embedded in 1% agarose. The samples were postfixed with 1% osmium acid in 0.1 M phosphate buffer solution (pH 7.4) for 2 h at room temperature and rinsed several times with 0.1 M phosphate buffer (pH 7.4). After dehydration with a series of graded ethanol solutions, the samples were dried in a critical point dryer and finally coated with gold by a sputter coater for SEM observation.

For TEM, Live MACS/VNP and LNT MACS/VNP were prefixed in 2.5% isopropanol at 4 °C for 2–4 h and washed with 0.1 M phosphate buffer (pH 7.4). Then, the samples were suspended and embedded in 1% agarose. The samples were postfixed with 1% osmium acid in 0.1 M phosphate buffer solution (pH 7.4) for 2 h at room temperature and rinsed several times with 0.1 M phosphate buffer (pH 7.4). After postfixation, they were dehydrated with a series of graded ethanol, and the sample was embedded in Epon812 and polymerized in an oven at 60 °C for 48 h. The resulting sample blocks were sectioned with an ultramicrotome to a thickness of 60–80 nm and doubly stained with 2% uranyl acetate and 2.6% lead citrate for TEM observation.

For the detection of ROS and nitric oxide (NO) production in macrophages after coincubation with the strains, the ROS assay kit (Solarbio, CA1420) and NO assay kit (Beyotime, S0021S, Shanghai, China) were used according to the manufacturers’ instructions. Flow cytometry (BD, Canto II, NJ, USA) was used to compare changes in cell size by comparing the forward scatter (FSC) values at the same voltage and changes in the internal structure of different cells by comparing the side scatter values at the same voltage. For the pathogenicity assay of cells in vivo, Live MACS or LNT MACS cells were injected subcutaneously (1 × 10^6^ cells per mouse) into the right flanks of BALB/c mice. Lump formation was observed every 1–2 days.

For fluorescence microscopy observation, Live MACS, Live MACS/VNP cells and LNT MACS/VNP cells were fixed with 4% paraformaldehyde for 30 min and permeabilized using 0.5% Triton X-100 (Sigma–Aldrich, 648462, St. Louis, MO, USA). After three washes with PBST, Alexa Flour 488 phalloidin (Beyotime, C2201S) was added and incubated for 1 h before staining with 4’,6-diamidino-2-phenylindole DAPI (Beyotime, C1005). VNP-RFP strains were self-expressed with RFP for intracellular tracing. Images were acquired using fluorescence microscopy (Carl Zeiss, Axioplan 2, Oberkohen, Germany).

### Quantitative real-time PCR

Total RNA was isolated using the TRIzol (Vazyme, R401-01-AA) extraction method and reverse transcribed using the ReverTra Ace qPCR RT Kit (Toyobo Life Science). The genes were amplified using AceQ qPCR SYBR Green Master Mix (Vazyme, Q221-01) according to the manufacturer’s instructions. The PCR primer sequences (see Supplementary Table 1) were designed using PrimerBank (https://pga.mgh.harvard.edu/primerbank/) and synthesized by Sangon Biotechas. Quantitative real-time PCR was performed using the Applied Biosystems StepOnePlus Real-Time PCR System.

### Intramacrophage VNP viability and release detection

Due to the self-protection mechanism of the VNP strain, a certain level of biological activity is still maintained inside macrophages. To detect the number of VNP strains effectively loaded in macrophages, the LNT MACS/VNP cell number was detected with the help of a cell counter, and the cells were subsequently lysed with 0.5% Triton X-100 at room temperature. The lysate was diluted and applied to LB agar plates supplemented with kanamycin and incubated overnight at 37 °C. The number of viable bacteria loaded in the cells was counted.

To detect the release of cells to the intracellular strain, the prepared Live MACS/VNP-RFP and LNT MACS/VNP-RFP cells were added to 96-well plates (1 × 10^4^ cells/200 μL/well), and the change in RFP fluorescence intensity (excitation light: 550 nm, emission light: 585 nm) within the pore size was determined by a multimode plate reader. The total fluorescence intensity increased with the release and proliferation of intracellular strains.

The *Salmonella*-specific type III secretion system enables specific induction of target protein expression only in macrophages, and we previously screened for potent promoters (sifB) under this system.^33^ The strains were monitored in macrophages using *Salmonella*-specific antibodies (Targetpharma, Nanjing, China). Fluorescence microscopy (Carl Zeiss) proved that intracellularly active bacteria expressed RFP. After obtaining LNT MACS/VNP cells incubated in vitro for different times (0/2/4 h), they were fixed using 4% paraformaldehyde and subsequently photographed by TEM as described above to detect the release and proliferation of intracellular strains. The prepared LNT MACS/VNP cells were cultured in culture medium supplemented with different concentrations of lactate, and the number of bacteria in the supernatant recovered from solid antibiotic resistance plates was used to determine the released intracellular strains at different time points.

### Morphology and infective infiltration ability of the strains

After obtaining LNT MACS/VNP cells, the cells were lysed using 0.5% Triton X-100 to release the intracellular strain. The strains (released VNP) in the supernatant were collected and cultured for 6 h in LB liquid medium for amplification. A normal VNP strain (Normal-VNP) was used as a control. The two kinds of VNP strains (Released-VNP, Normal-VNP) were prefixed in 2.5% isopropanol and subsequently used for SEM filming. To test the ability of the strain to infect tumor cells, H22 cells were infected with different VNP at an MOI of 100 for 1 h. After infection, the cells were washed with PBS and then treated with 50 μg/mL gentamicin for 30 min to remove residual strains. Cells were lysed with 0.5% Triton X-100, and the number of internalized VNP was determined by plating dilutions of the cell lysates on LB plates. Indirect coculture experiments of cells with the aid of Transwell were performed as previously described.^25^

### Bacterial growth assays

The growth conditions of different strains in LB medium were monitored using Bioscreen C (OY Growth Curves Ab Ltd, Finland). In brief, the VNP strains, activated after two subcultures, were adjusted to an OD600 of 1.0. Then, 10 μL of the bacterial suspension was added to 1 mL of LB medium. After thorough mixing, 300 μL of the solution was added to the wells of the Bioscreen C microplate. The microplate was incubated at 37 °C for 30 h, with continuous OD600 measurements taken at 30-min intervals.

### Detection of apoptosis

H22 cells (1.0–3.0 × 10^5^) were plated onto 12-well plates and allowed to adhere to the wall for 6 to 8 h of incubation. Afterwards, the cells were cocultured with the gathered normal-VNP or released VNP strains for 4 h at an MOI of 100. All of the cells in the plate were removed, cleaned, and washed and resuspended in 100 μL of 1 × binding buffer before being stained with 1 μg of laboratory-made annexin V protein (conjugate APC) and kept on ice in the dark for 30 min. Before flow cytometry analysis, 1 μL of propidium iodide (25 g/mL) was added to all samples and gently mixed.

### Cell surface ligand and chemokine receptor assay

Cell adhesion molecules, e.g., CD11b, and chemokine receptors, e.g., CCR2, play crucial roles in macrophage anchoring at the tumor site. Total proteins from Live MACS, Live MACS/VNP and LNT MACS/VNP cells were collected for Western blot analysis as previously described.^56^ The protein concentration was determined by BCA assay (Beyotime, P0012). Equal amounts of total protein were separated by SDS–PAGE and transferred onto PVDF membranes. The membranes were blocked in PBST containing 5% skim milk for 1 h at room temperature, and the membranes were then incubated at 4 °C overnight with primary antibodies, including CD11b antibody (ABclonal, A1581, Wuhan, China), CCR2 antibody (Proteintech, 16153-1-AP, Wuhan, China) and GAPDH (ABclonal, M20006), followed by incubation with HRP-labeled secondary anti-rabbit IgG antibody (CST, 7074). Protein band intensity was quantified by ImageJ software.

The expression of CD11b and CCR2 on the cell surface was analyzed by fluorescence microscopy and flow cytometry. For confocal microscopy analysis, Live MACS, Live MACS/VNP and LNT MACS/VNP cells were suspended in 1% BSA and incubated with protein-specific antibodies. After 2–3 washes with PBST, FITC-labeled fluorescent secondary antibody (Absin, abs20004, Shanghai, China) was used to identify the primary antibody. Fluorescence microscopy (Carl Zeiss, Axioplan 2, Oberkohen, Germany) was used to observe the labeling. For flow cytometry analysis, Live MACS, Live MACS/VNP and LNT MACS/VNP cells were suspended in cell staining buffer and stained with CD11b-APC (BD, 553312) and CCR2-AF647 (Biolegend, clone SA203G11) for 30 min at 4 °C in a light-proof environment. The precipitate was collected by centrifugation and washed 1–2 times with PBS. Cell surface fluorescence was analyzed by flow cytometry after resuspension in PBS.

### Animal model

All procedures were conducted in compliance with all the relevant ethical regulations and were approved by the Nanjing University Institutional Animal Care and Use Committee. BALB/c (6 weeks, female) mice were purchased from Changzhou Cavens Animals Corporation. H22 tumor cells were injected subcutaneously (1 × 10^6^ cells per mouse) into the right flanks of BALB/c mice. When tumors grew to 80–120 mm^3^, LNT VNP strains (1.0 × 10^7^ cells in each mouse) and cells (4.0 × 10^6^ cells in each mouse), including LNT MACS and LNT MACS/VNP, were administered only once through the caudal vein. LNT MACS + VNP (a simple mixture of the two) was used as an uncamouflaged control for LNT MACS/VNP, and the number of cells and bacteria used was referenced as described above. Tumor volumes (V, mm^3^) were determined using the formula V = a^2^b × 0.52. Here, “a” represents the minor diameter and “b” represents the major diameter. Tumors were measured using calipers three to four times each week.

Tumor volume doubling time was calculated using the following equation:

$${\rm{DT}}=\frac{{\rm{t}}\times \log 2}{\log \frac{{{\rm{V}}}_{{\rm{t}}}}{{{\rm{V}}}_{0}}}$$

where “t” represents the time interval in days between observations before and after tumor assessment, “Vt” stands for the tumor volume at time “t”, and “V0” represents the initial tumor volume at the starting time.

A retro-orbital puncture was used to draw blood. In preparation for ELISA and blood biochemical index analysis, blood serum was collected and cryopreserved at −80 °C. Wuhan Servicebio Corporation ran regular blood tests, analyzed blood biochemistry, and stained heart, liver, spleen, lung, and kidney sections with H&E. Tumor-bearing mice were euthanized when unfavorable side effects (pain, apathy, or a necrotic tumor) were observed or the humane goal (tumor weight corresponding to 10% of mouse body weight) was reached. For the detection of titers of free strains in peripheral blood, VNP, and LNT MACS/VNP were administered to tumor-bearing mice. Then, peripheral blood was taken from the mice after different time points followed by a brief transient separation, and the supernatant was spread on solid plates containing the resistance.

### In vivo biodistribution

LNT MACS and LNT MACS/VNP cells were prepared, and 1.0 × 10^7^ LNT VNP, 4.0 × 10^6^ LNT MACS/VNP cells and 4.0 × 10^6^ LNT MACS mixed with 1.0 × 10^7^ LNT VNP (LNT MACS + VNP) suspended in 150 μL of saline were administrated into H22 subcutaneous tumor model mice *via* the tail vein. Tumors and other organs (including the heart, liver, spleen, lung, and kidneys) were dissected and lysed with a tissue pulverizer after the mice were slaughtered at predetermined periods. To determine the bacterial distribution, dilute the tissue lysate to an appropriate concentration and then plate it on LB agar plates to form individual bacterial colonies. Count the colonies the next day to calculate the bacterial distribution.

LNT MACS and LNT MACS/VNP-LuxCDABE cells were extracted as previously described, and then treated with the near‐infrared (NIR) lipophilic carbocyanine tracer DiR (Abbkine, BMD0074, Wuhan, China) for 30 min to produce DiR-tagged cells. DiR-labeled LNT cells were treated with 4% paraformaldehyde for 1 h to denature proteins as the control group. LNT MACS mixed with LNT VNP-LuxCDABE (LNT MACS + VNP-LuxCDABE), LNT MACS/VNP- LuxCDABE cells and paraformaldehyde-fixed LNT MACS mixed with LNT VNP-LuxCDABE (Fixed LNT MACS + VNP-LuxCDABE) suspended in 150 μL of saline were administrated into H22 subcutaneous tumor model mice *via* the tail vein. The luminescence intensity of LuxCDABE and fluorescent signals of DiR in the tumors of mice after different treatments were identified using an in vivo imaging system (IVIS) imager (PerkinElmer, Waltham, MA, USA).

### Isolation of Peritoneal Neutrophils

1 ml of nutritious broth was administered intraperitoneally 6 h before the isolation of mouse peritoneal neutrophils to stimulate peritoneal neutrophil maturation. Neutrophils were isolated by gradient centrifugation and coincubated with LNT MACS/VNP and a simple mixture of LNT MACS and LNT VNP (in the proportion of cell number 1:1) for 60 min. The free NETs were stained with nucleic acid dye DAPI, and the absorbance was measured using a microplate reader.

### Enzyme-linked immunosorbent assay (ELISA)

After administration, the mice were bled for blood (~200 μL) and collected into tubes. Whole blood was allowed to stand at room temperature for 30 min and then centrifuged at 3000 rpm for 15 min. The supernatant was collected for IL-6/IL-10, which are positively correlated with the degree of inflammation occurring in the body,^12^ cytokine assays by mouse IL-6 ELISA kits (BYabscience, BY-EM220188, Nanjing, China), and IL-10 ELISA kits (Liankebio, EK210, Hangzhou, China), separately, according to the manufacturers’ instructions. For the intratumor cytokine assay, the tumor was mixed with tissue lysate (absin, abs9225) and homogenized with a tissue homogenizer. The supernatant was centrifuged and utilized for detecting TNFα and IFNγ cytokines with mouse TNFα ELISA kits (BYabscience, BY-EM220852) and IFNγ ELISA kits (BYabscience, BY-EM220140). For the detection of ROS produced by cells, after 60 min of coincubation, the neutrophils were coincubated with LNT MACS/VNP or a simple mixture of LNT MACS + LNT VNP (the cell number was added at 1:1) for 60 min. The supernatant was collected by centrifugation and used for ROS detection by mouse ROS ELISA kits (YIFEIXUE, YFXEM00485, Nanjing, China).

### Flow cytometry

After the mice had been thoroughly anesthetized, the eyeball was removed to retrieve whole blood, and blood anticoagulant was added. A peripheral blood lymphocyte isolation kit (Solarbio, P8620) was used to collect peripheral blood lymphocytes. To obtain single-cell suspensions, cell clumping was removed using a 40-μm cell strainer. The cells were stained with the following anti-mouse antibodies: CD4-APC (BD, 553051), CD25-BV421 (BD, 562606), CD127-PE (BD, 552543), CD11b-APC (BD, 553312), Ly6G-BV421 (BD, 562737), and CD62L-PE (BD, 553151). Specifically, the collected peripheral blood lymphocytes were resuspended in 1% BSA in Hanks buffer, followed by the addition of the CD4, CD25 and CD127 antibodies described above to detect Treg cells in mouse peripheral blood and CD11b, Ly6G and CD62L antibodies to detect activated neutrophils in mouse peripheral blood. After incubation for 30 min at 4 °C, the cells were washed 2–3 times with buffer to remove unbound antibodies and assayed on the instrument.

For detection of microenvironmental changes within tumors, tumor-bearing mice were sacrificed after different treatments, and the tumors were collected and then cultured in collagenase digestion medium for 60 min. To obtain single-cell suspensions, cell clumping was removed using a 40-μm cell strainer. The cells were stained with fixable viability dye (BD, 564407), incubated for 30–45 min at 4 °C protected from light, and then stained using the following anti-mouse antibodies: CD45-BV510 (BD, 740131), CD3-FITC (BD, 561801), CD8-PE-Cy7 (BD, 552877), CD4-PerCP-Cy5.5 (BD, 550954), CD86-PE (Invitrogen, 12-0862-82), CD11c-PE-Cy7 (BD, 558079), MHCII-FITC (BD, 562009), F4/80-APC (BD, 566787), F4/80-BV421 (BD, 565411), CD11b-FITC (BD, 561684), and Ly6G-PerCP-Cy5.5 (BD, 560602). Intracellular proteins, including CD206-APC (Invitrogen, 17-2061-82), Foxp3-PE (BD, 563101), and GzmB-APC (Invitrogen, 17-8898-82), were stained after using a fixative reagent (BD, 562574) with membrane breaking function. The BD FACS Canto II was used for flow cytometric analyses.

### Statistics analysis

Protein band intensity was quantified by ImageJ software. GraphPad Prism software version 7 (GraphPad Software) was used to analyze the data. Student’s *t* test was used to compare two groups. One-way analysis of variance (ANOVA) was used to compare more than two groups, followed by Dunnett’s multiple comparisons test. The data are presented as the mean ± SD.

### Supplementary information

Supplemental Material

## Data Availability

All data reported in this paper are available upon request.
