# Supplementary material for: Camouflaging attenuated Salmonella by cryo-shocked macrophages for tumor-targeted therapy
Source: Signal Transduct Target Ther. 2024 Jan 10;9:14. doi: 10.1038/s41392-023-01703-1 (PMC10776584; doi:10.1038/s41392-023-01703-1)
Supplement: Supplementary file 1 — Supplemental Material [file 41392_2023_1703_MOESM1_ESM.docx]

Supplementary Materials for

**Camouflaging attenuated *Salmonella* by cryo-shocked macrophages for tumor-targeted therapy**

Leyang Wu^1,2,3^, Zengzheng Du^1^, Lin Li^1^, Liyuan Qiao^1^, Shuhui Zhang^1^, Xingpeng Yin^1^, Xiaoyao Chang^1^, Chenyang Li^1^, Zichun Hua^1,2,3,4^

Correspondence to: zchua@nju.edu.cn

**This PDF file includes:**

Figures. S1 to S10

Tables S1

**Supplementary Figure captions and legends**


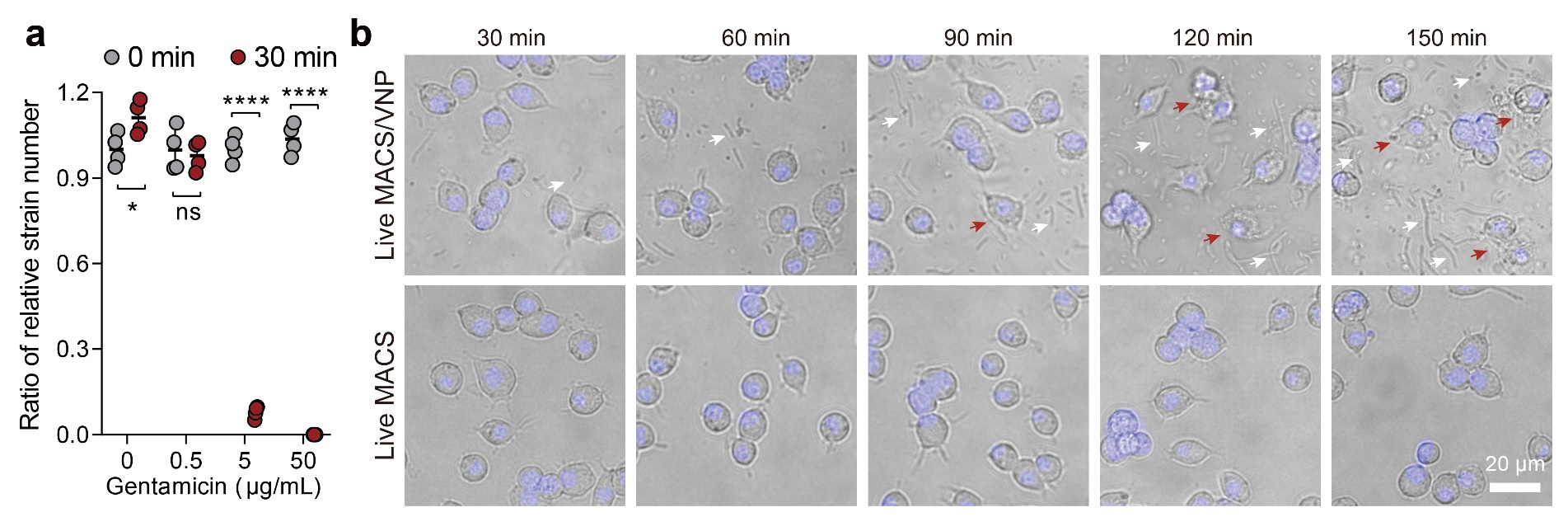


**Figure S1** **Preparation of** **Live MACS/VNP.**

**a** The VNP strains was inoculated with 1 mL of DMEM supplemented with different concentrations of gentamicin (0/0.5/5/50 μg/mL) and the number of bacteria was counted after incubation for 30 minutes (*n* = 4).

**b** Bright-field pictures of MACS after coculture with the VNP strain at different time points (30-150 min, MOI = 20). Hoechst marks nuclei (blue). White arrows indicate the VNP strains, and red arrows indicate the broken cells. Bacteria become more numerous due to proliferation, and the percentage of broken cells increases over time. Scale bar = 20 μm.

Data are reported as the mean ± SD. All data are representative of two independent experiments. n.s. = not significant, **p* < 0.05, *****p* < 0.0001.


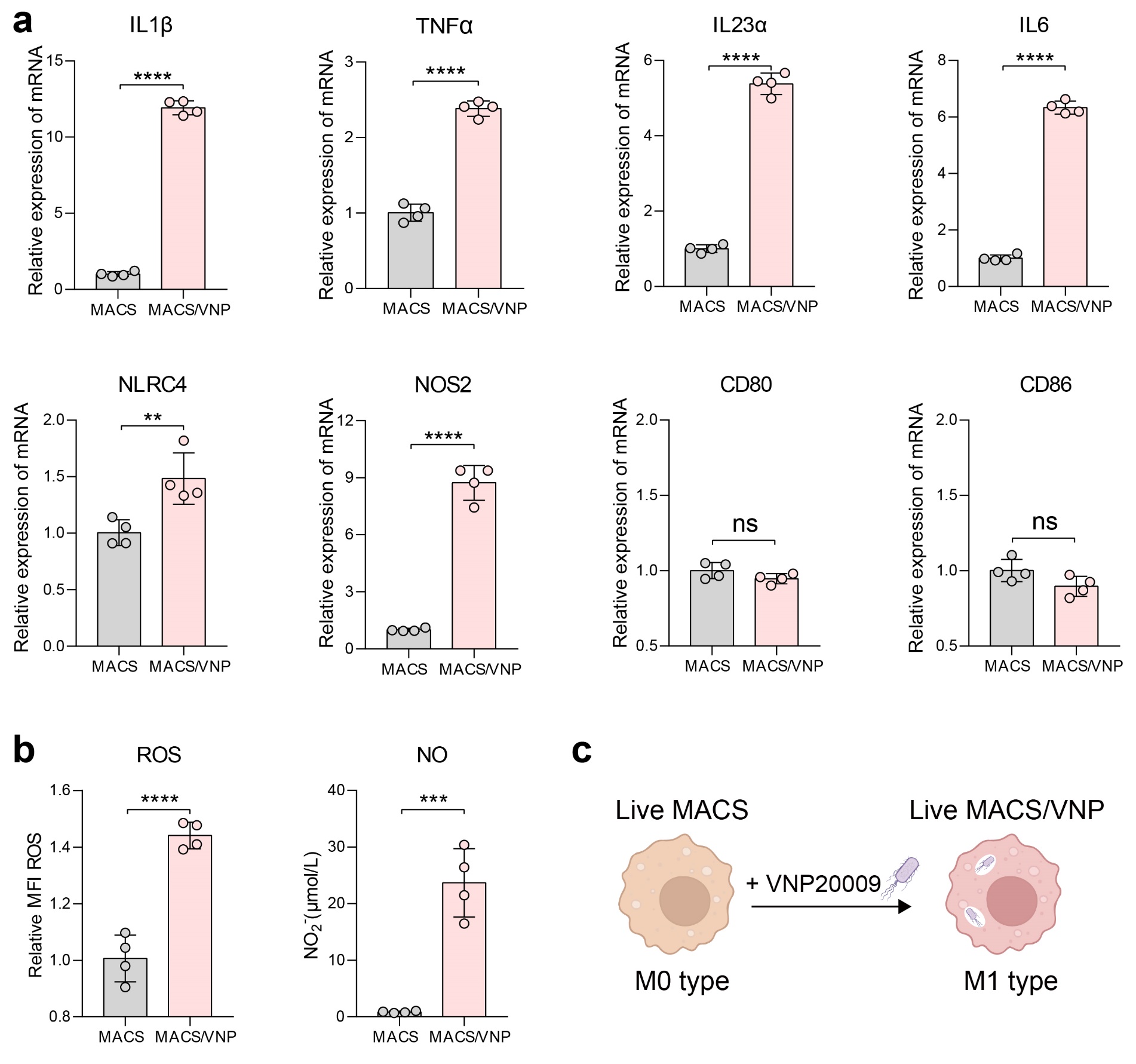


**Figure S2 Live MACS/VNP cells shift to a proinflammatory M1 phenotype.**

**a** Detection of changes in the expression levels of M1-type marker genes in Live MACS and Live MACS/VNP (*n* = 4).

**b** Detection of intracellular ROS (left) and NO (right) in Live MACS and Live MACS/VNP cells (*n* = 4).

**c** Schematic diagram of the changes in macrophage responses induced by *Salmonella*. Bacteria cause a range of proinflammatory responses in macrophages, including upregulation of cytokine gene expression and increased production of reactive oxygen species.

Data are reported as the mean ± SD. All data are representative of two independent experiments. n.s. = not significant, ***p* < 0.01, ****p* < 0.001, *****p* < 0.0001.


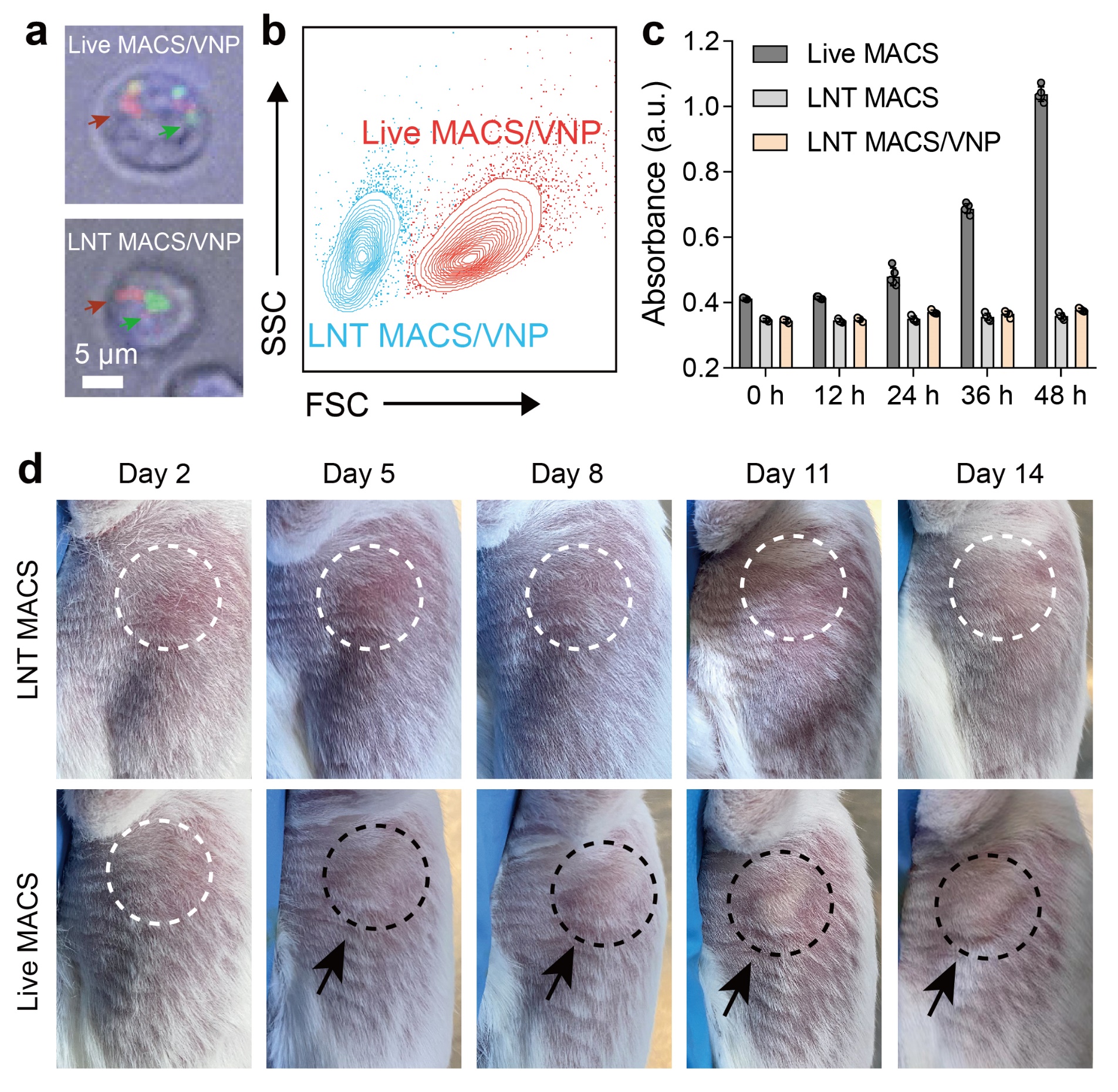


**Figure S3 LNT MACS/VNP cells lose proliferative activity.**

**a** Bright-field pictures of Live-MACS/VNP and LNT-MACS/VNP cells loaded with two different engineered strains (VNP-RFP and VNP-GFP strains). Hoechst marks nuclei (blue). Red arrows indicate the VNP-RFP strains (strain expressing red fluorescent protein). Green arrows indicate the VNP-GFP strains (strain expressing green fluorescent protein). Scale bar = 5 μm.

**b** Flow cytometry analysis of Live MACS/VNP and LNT MACS/VNP cells. FSC, forward scatter; SSC, side scatter. The FSC values confirmed the reduced cell size of LNT MACS/VNP cells, and the SSC values indicated that the internal structure of LNT MACS/VNP cells was maintained.

**c** Cell viability analysis of Live MACS/VNP and LNT MACS/VNP cells by CCK8 assay (*n* = 5). a.u., arbitrary unit.

**d** In vivo bioactivity analysis of Live MACS and LNT MACS cells. Black arrows point to areas where masses appear due to continued cell proliferation.

Data are reported as the mean ± SD. All data are representative of two independent experiments.


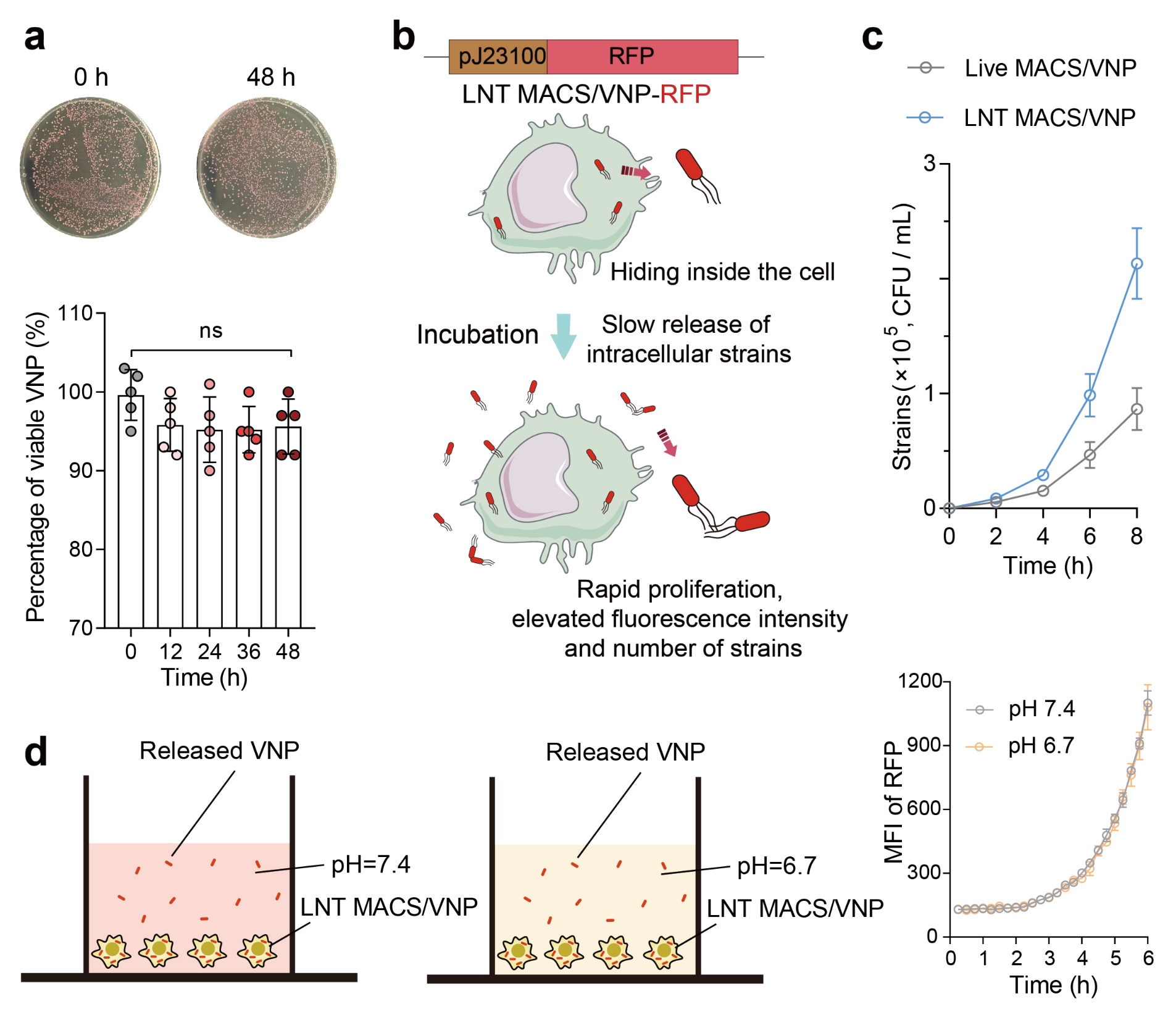


**Figure S4 The VNP strain is resistant to cryo-shocking with liquid nitrogen and maintains its biological activity after environmental recovery.**

**a** Survival ratios of VNP strains after different times (0-48 h) of liquid nitrogen cold treatment. The engineered strain VNP-RFP was used for more convenient observation (*n* = 5).

**b** Schematic diagram of the detection of VNP strains released from the cell. The released VNP-RFP strains continue to proliferate and express red fluorescence protein, so the total fluorescence intensity in the same volume is positively correlated with the number of strains, which also implies a higher titer of strains in the supernatant.

**c** Detection of strain titers in the supernatants of Live MACS/VNP and LNT MACS/VNP cells at different time points (0-8 h) (*n* = 5).

**d** The prepared LNT MACS/VNP cells were incubated in medium with different pH values (7.4 and 6.7) adjusted by dilute hydrochloricacid (left), and the change in RFP fluorescence intensity of cell culture plates was monitored in real time (right) (*n* = 3).

Data are reported as the mean ± SD. All data are representative of two independent experiments. n.s. = not significant.


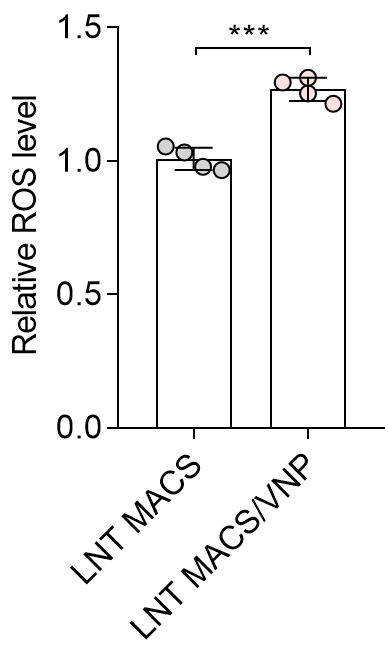


**Figure S5** Comparison of relative ROS levels between LNT MACS and LNT MACS/VNP (*n* = 4). Data are reported as the mean ± SD. All data are representative of two independent experiments. ****p* < 0.001.


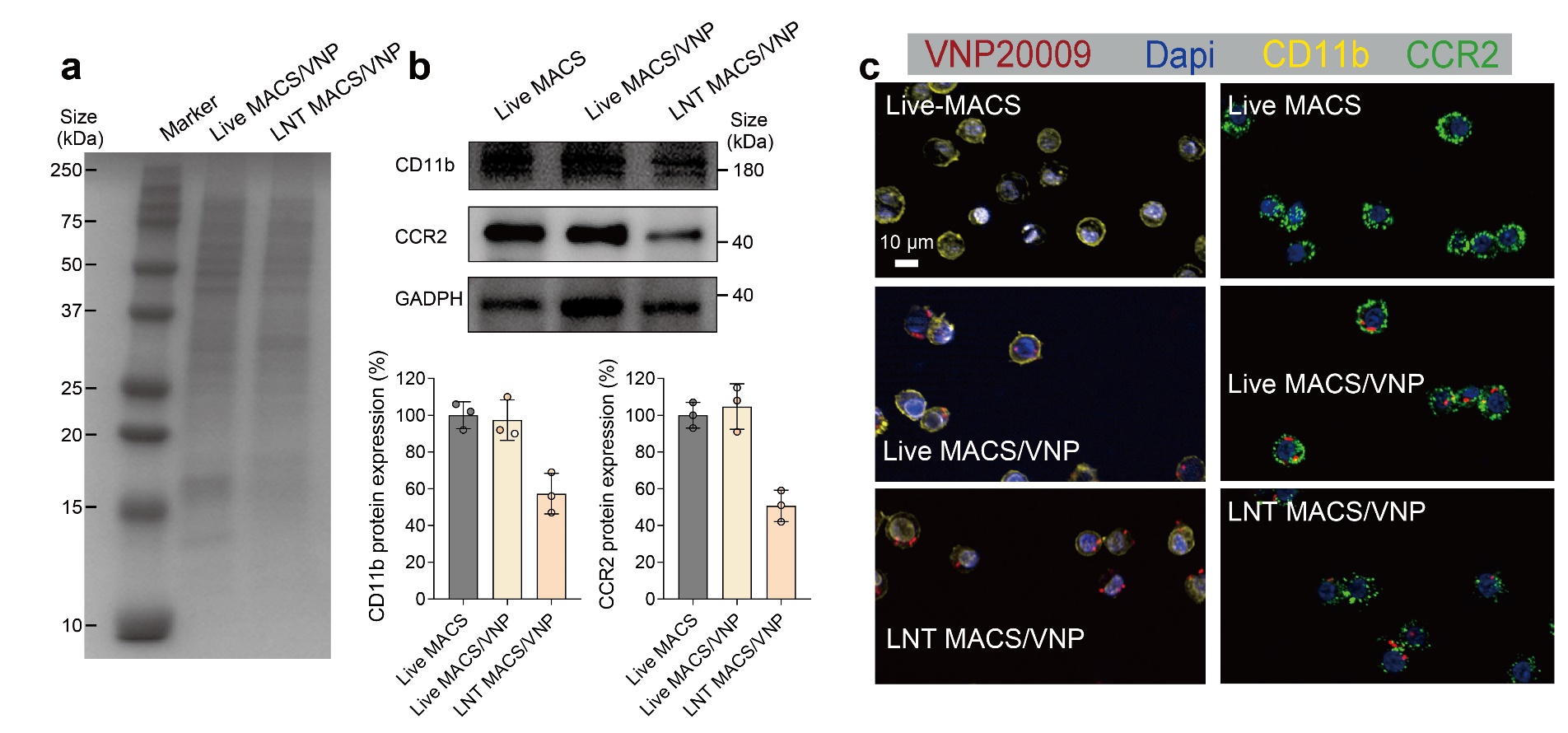


**Figure S6 CD11b and CCR2 expression in strain-loaded LNT cells.**

**a** Sodium dodecyl sulfate polyacrylamide gel electrophoresis (SDS‒PAGE) of whole-cell lysate proteins obtained from Live MACS-VNP and LNT-MACS-VNP cells.

**b** Representative western blotting (top) and quantification (bottom) of CD11b and CCR2 in LNT MACS/VNP cells (*n* = 3).

**c** CD11b and CCR2 expression in Live MACS, Live MACS/VNP and LNT MACS/VNP cells analyzed by confocal microscopy. Scale bar = 10 μm. Data are reported as the mean ± SD.


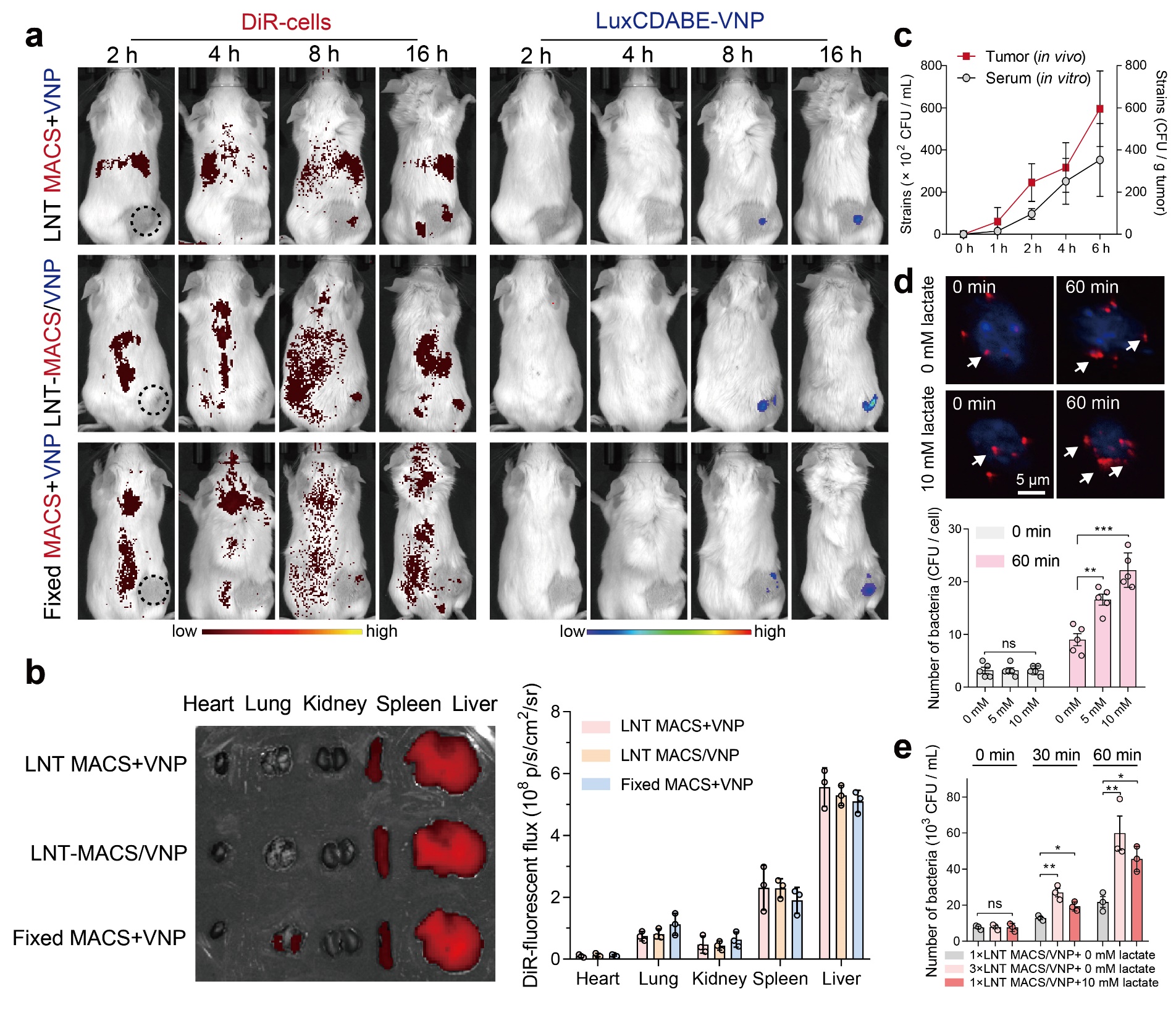


**Figure S7 LNT MACS/VNP cells achieve efficient and specific release of intracellular bacteria to tumors.**

**a** Tumor trafficking and biodistribution of DiR-labeled LNT MACS cells mixed with LNT VNP-LuxCDABE strains, LNT MACS/VNP-LuxCDABE cells, or DiR-labeled fixed LNT MACS cells mixed with LNT VNP-LuxCDABE strains (4.0 × 10^6^ cells per mouse) in H22 tumor xenograft models were analyzed by Live animal imaging at different time points after intravenous administration.

**b** *In vivo* imaging system (IVIS) image of typical organs and relative fluorescence intensities of the mice 8 h post injection of DiR-labeled LNT cells (*n* = 3).

**c** Determination of the number of free strains released from LNT MACS/VNP cells cultured in serum and intratumoral titers of strains at different time points after LNT MACS/VNP cell administration (*n* = 5).

**d** Comparison of the number of intracellular strains in LNT MACS/VNP cells under the addition of 0/5/10 mM lactate (bottom) and representative fluorescence photos (top) (*n* = 5).

**e** Determination of the number of supernatant-free strains in different LNT MACS/VNP cell culture media (1×LNT MACS/VNP: approximately 3 strains loaded per cell, 3×LNT MACS/VNP: approximately 9 strains loaded per cell) and 1×LNT MACS/VNP cell culture medium supplemented with 10 mM lactate (*n* = 3).

Data are reported as the mean ± SD. Statistics were calculated using the two-tailed, unpaired Student’s t test with Welch’s correction. n.s. = not significant, **p* ≤ 0.05, ***p* ≤ 0.01, ****p* < 0.001.


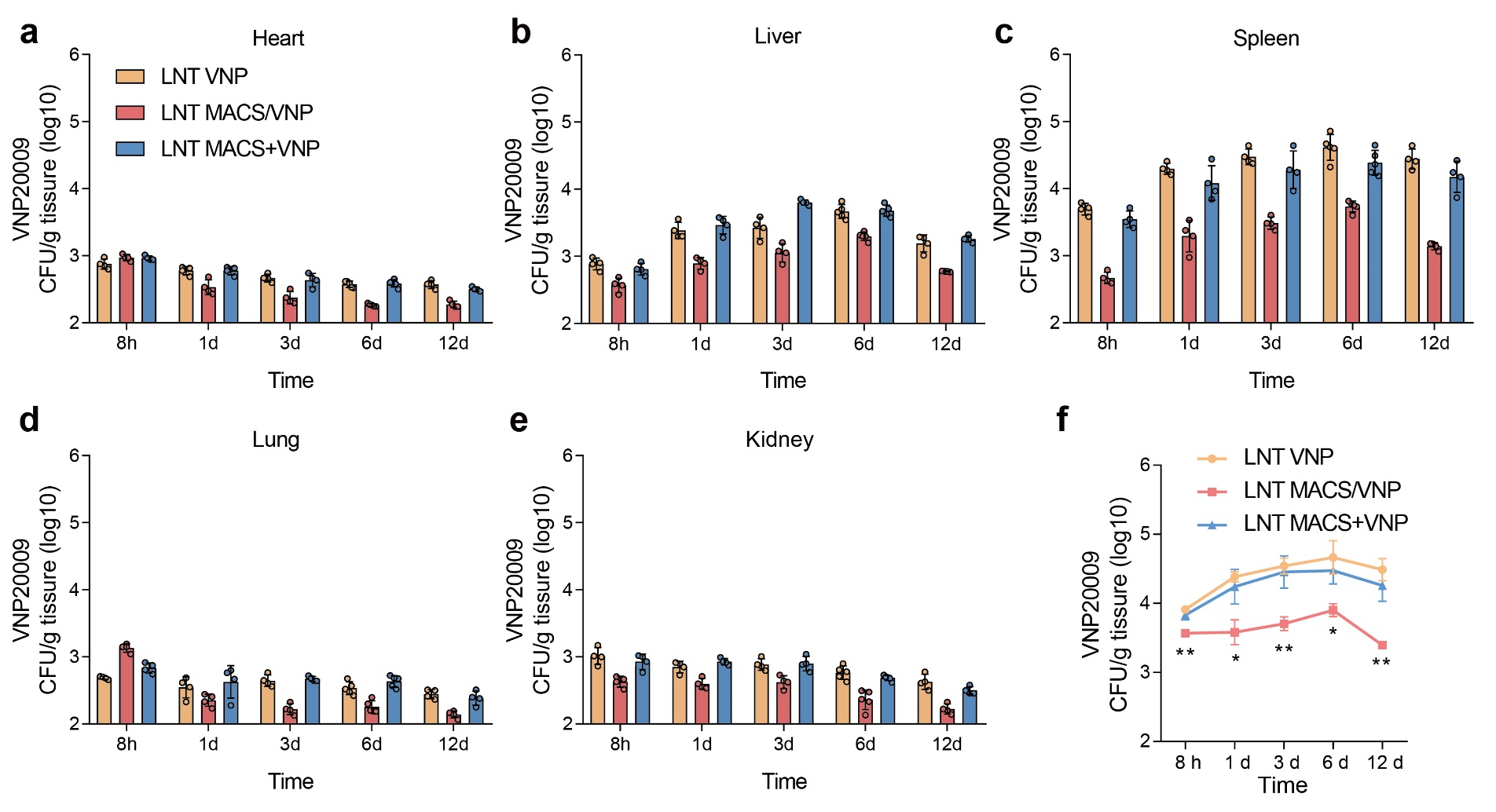


**Figure S8 LNT MACS/VNP cells reduce bacterial off-targeting in normal organs.**

**a-e** The VNP strain titers in the heart (a), liver (b), spleen (c), lung (d) and kidney (e) were determined at specific time points after the administration of LNT VNP, LNT MACS/VNP cells and LNT MACS+VNP cells (*n* = 4 or 5).

**f** The relationship between time postinjection and bacterial number within the normal organs (including heart, liver, spleen, lung and kidney, *n* = 4 or 5).

Data are reported as the mean ± SD. All data are representative of two independent experiments. Statistics were calculated using the two-tailed, unpaired Student’s t test with Welch’s correction. **p* ≤ 0.05, ***p* ≤ 0.01.


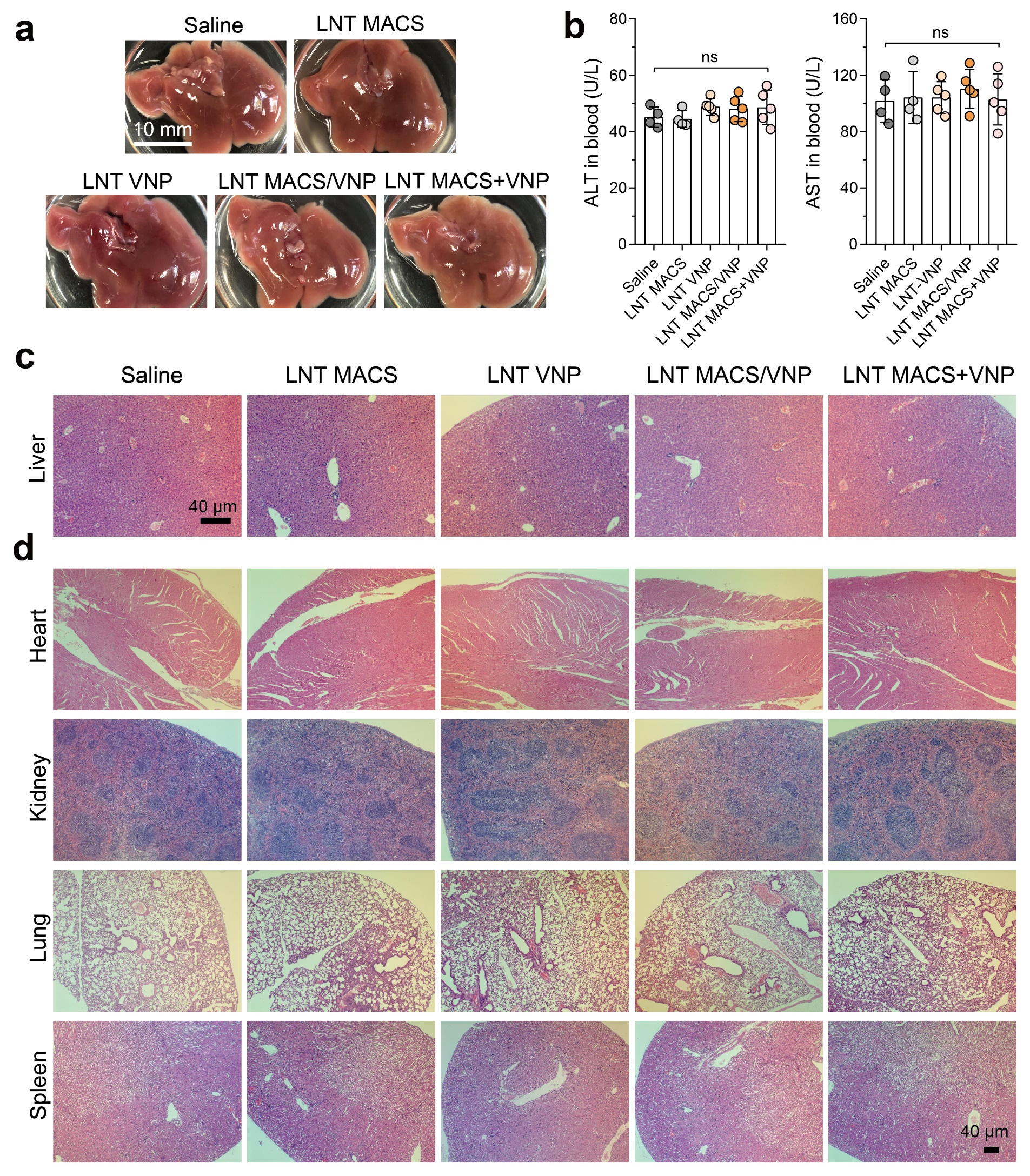


**Figure S9 LNT MACS/VNP cells show no detectable chronic toxicities.**

**a** Representative pictures of the liver in each group of mice 12 days after administration. Scale bar = 10 mm.

**b** Serum alanine transaminase (ALT) and aspartate transaminase (AST) levels 12 days after different treatments (*n* = 4 or 5).

**c** A close-up view of representative H&E staining of liver sections (scale bar = 40 µm).

**d** A close-up view of representative H&E staining of heart, kidney, lung and spleen sections (Scale bar = 40 µm).

Data are reported as the mean ± SD. All data are representative of two independent experiments. n.s. = not significant.


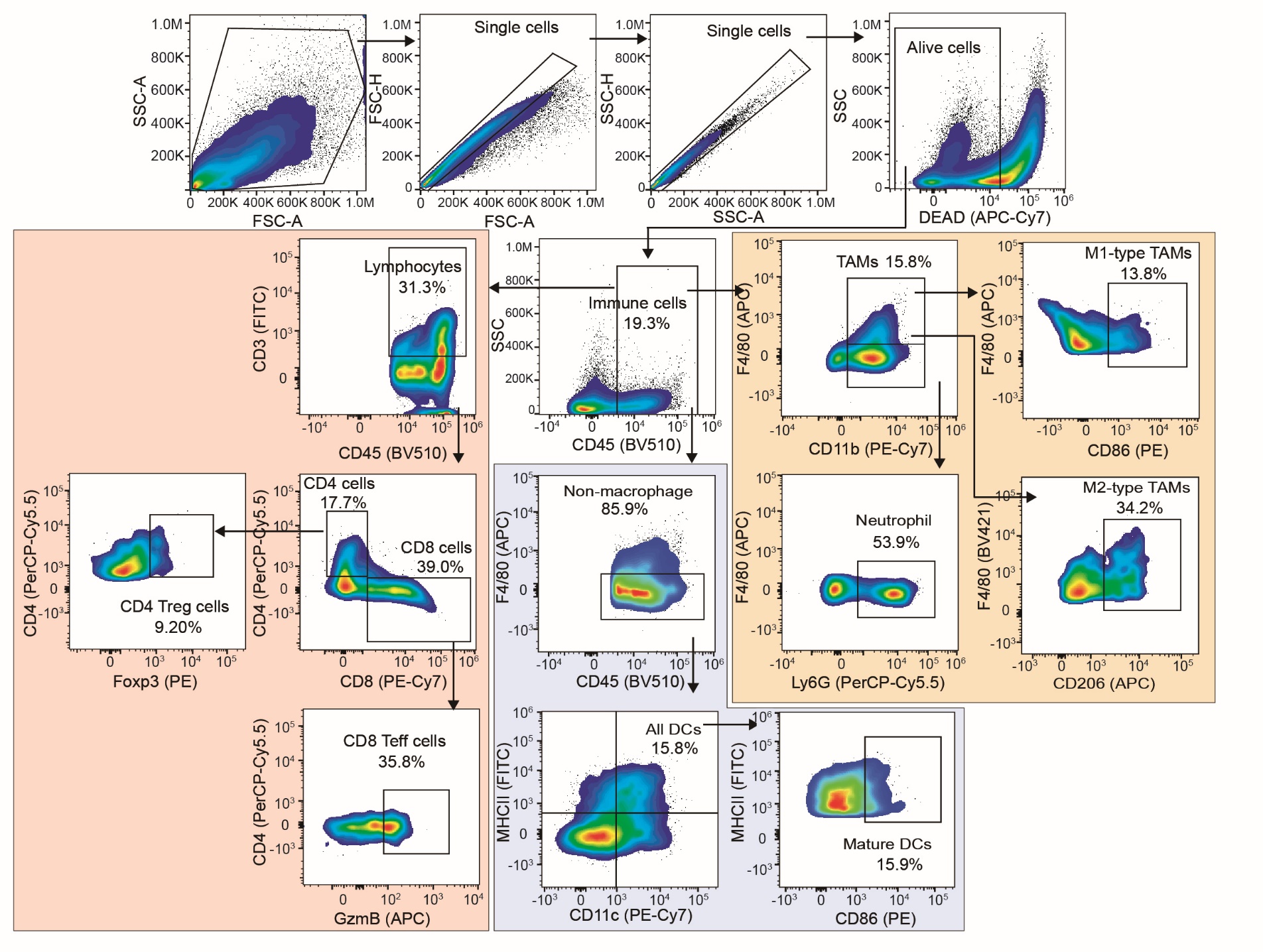


**Figure S10** Representative gating strategy to identify lymphocytes, dendritic cells (DCs), neutrophils and tumor-associated macrophages (TAMs) with different phenotypes.

**Table S1** Primer sequences for RT‒PCR.

| Primer | Forward (5'-3') | Reverse (5'-3') |
| --- | --- | --- |
| β-actin | GCA CCA CAC CTT CTA CAA TGA G | TTG GCA TAG AGG TCT TTA CGG A |
| IL1β | GCA ACT GTT CCT GAA CTC AAC T | ATC TTT TGG GGT CCG TCA ACT |
| TNF-α | CCC TCA CAC TCA GAT CAT CTT CT | GCT ACG ACG TGG GCT ACA G |
| IL23α | AAT AAT GTG CCC CGT ATC CAG T | GCT CCC CTT TGA AGA TGT CAG |
| IL6 | TAG TCC TTC CTA CCC CAA TTT CC | TTG GTC CTT AGC CAC TCC TTC |
| NLRC4 | ATC GTC ATC ACC GTG TGG AG | GCC AGA CTC GCC TTC AAT CA |
| NOS2 | GTT CTC AGC CCA ACA ATA CAA GA | GTG GAC GGG TCG ATG TCA C |
| CD68 | TGT CTG ATC TTG CTA GGA CCG | GAG AGT AAC GGC CTT TTT GTG A |
| CD80 | TGC TGC TGA TTC GTC TTT CAC | GAG GAG AGT TGT AAC GGC AAG |
| CD86 | TGT TTC CGT GGA GAC GCA AG | TTG AGC CTT TGT AAA TGG GCA |
